# Tick-Box for 3′-End Formation of Mitochondrial Transcripts in Ixodida, Basal Chelicerates and *Drosophila*

**DOI:** 10.1371/journal.pone.0047538

**Published:** 2012-10-15

**Authors:** Matteo Montagna, Davide Sassera, Francesca Griggio, Sara Epis, Claudio Bandi, Carmela Gissi

**Affiliations:** 1 Dipartimento di Scienze Veterinarie e Sanità Pubblica, Università degli Studi di Milano, Milano, Italy; 2 Dipartimento di Bioscienze, Università degli Studi di Milano, Milano, Italy; The Centre for Research and Technology, Hellas, Greece

## Abstract

According to the tRNA punctuation model, the mitochondrial genome (mtDNA) of mammals and arthropods is transcribed as large polycistronic precursors that are maturated by endonucleolytic cleavage at tRNA borders and RNA polyadenylation. Starting from the newly sequenced mtDNA of *Ixodes ricinus* and using a combination of mitogenomics and transcriptional analyses, we found that in all currently-sequenced tick lineages (Prostriata, Metastriata and Argasidae) the 3′-end of the polyadenylated *nad1* and *rrnL* transcripts does not follow the tRNA punctuation model and is located upstream of a degenerate 17-bp DNA motif. A slightly different motif is also present downstream the 3′-end of *nad1* transcripts in the primitive chelicerate *Limulus polyphemus* and in *Drosophila* species, indicating the ancient origin and the evolutionary conservation of this motif in arthropods. The transcriptional analyses suggest that this motif directs the 3′-end formation of the *nad1*/*rrnL* mature RNAs, likely working as a transcription termination signal or a processing signal of precursor transcripts. Moreover, as most regulatory elements, this motif is characterized by a taxon-specific evolution. Although this signal is not exclusive of ticks, making a play on words it has been named “Tick-Box”, since it is a check mark that has to be verified for the 3′-end formation of some mt transcripts, and its consensus sequence has been here carefully characterized in ticks. Indeed, in the whole mtDNA of all ticks, the Tick-Box is always present downstream of *nad1* and *rrnL*, mainly in non-coding regions (NCRs) and occasionally within *trnL(CUN).* However, some metastriates present a third Tick-Box at an intriguing site - inside the small NCR located at one end of a 3.4 kb translocated region, the other end of which exhibits the *nad1* Tick-Box - hinting that this motif could have been involved in metastriate gene order rearrangements.

## Introduction

Chelicerates constitute a major lineage within Arthropoda and encompass taxa of evolutionary interest, such as the deep-branching lineage Xiphosura (including the living fossil *Limulus polyphemus*), and species of medical relevance, such as the Arachnida (e.g. ticks, mites, scorpions, spiders). Mitogenomic studies of chelicerates are thus conducted both to elucidate their evolutionary biology and to derive mitochondrial sequences for use in species identification. Ticks (Ixodida) are obligate blood-sucking ectoparasites that originated in early/middle Permian (300–260 million years ago, Mya) [1], [2], [3] and now parasitize a variety of terrestrial vertebrates [4], [5]. The approximately 870 described species of ticks are subdivided into three families: Argasidae, Ixodidae and Nuttalliellidae [6]. Ixodidae (hard ticks) can be divided in two morphological groups: Prostriata, including only the genus *Ixodes*, and Metastriata, including the remaining 12 genera [7]. Ticks can transmit a variety of pathogenic agents to humans and animals [4]. In particular, the sheep tick *Ixodes ricinus* (Linnaeus 1758), the most common blood-feeding ectoparasite in Europe, is the vector of Lyme disease and other bacteria, protozoa and viruses [8]. *I. ricinus* is also of particular interest in that it harbours a symbiont, “*Candidatus* Midichloria mitochondrii*”* [9], that resides in the intermembrane space of mitochondria. It can thus be considered a model of a three-levels relationship: the vertebrate host, the tick ectoparasite, and the intra-mitochondrial bacterium “*Candidatus* M. mitochondrii”.

Currently the complete mitochondrial genome (mtDNA) has been sequenced in 55 chelicerate species, including the living fossil *L. polyphemus*, whose gene order is considered to be ancestral for all arthropods [10], [11]. Chelicerate mitogenomes show several distinctive features compared to other arthropods: bizarre tRNA structures [12], [13], [14], [15]; unusual rRNAs [16], [17]; fast nucleotide substitution rate [18]; and extensive gene order rearrangements even between closely related species [17], [19], [20], [21], [22], [23], [24]. Indeed, among the 55 complete mtDNAs of chelicerates, the primitive gene order of *L. polyphemus* is shared only by two whip spiders from the order Amblypygi (*Phrynus* sp. and *Damon diadema,*), the mesothele spider *Heptathela hangzhouensis*, the scorpion *Uroctonus mordax*, and several tick species (suborder Ixodida) (see http://www.caspur.it/mitozoa).

Given the general interest in the Ixodida, we sequenced the complete mtDNA of the sheep tick *I. ricinus*. The comparison to other tick mtDNAs highlighted several oddities in the *nad1* and *rrnL* genes that prompted us to investigate the transcription of these genes in all major tick lineages (Prostriata, Metastriata and Argasidae). Therefore, we carried out 3′ RACE experiments in *I. ricinus*, and mapped the exact 3′-end of these transcripts in several other ticks, using thousands of available tick EST sequences and according to the strategy described in Gissi et al. [25].

In this paper, after a brief summary of the main features of the *I. ricinus* mtDNA, we describe the identification of a degenerate 17 bp sequence motif directing the 3′-end formation of *nad1* and *rrnL* transcripts in all major tick lineages. This motif represents an exception to the tRNA punctuation model, which predicts that arthropod mtDNA is transcribed in large polycistronic RNA precursors maturated through endonucleolytic cleavages and polyadenylation at sites immediately adjacent to tRNA genes [26], [27], [28]. Using genomics and transcriptional data, we also demonstrate the presence of a similar sequence motif, playing a similar function, downstream of the only *nad1* gene in the basal chelicerate *L. polyphemus* and in the model hexapod *Drosophila melanogaster*. Finally, we illustrate a possible evolutionary scenario of this motif from chelicerates to hexapods. Making a play on word, we have named this motif “Tick-Box”, since it is a “check mark” that has to be verified for the 3′-end formation of *nad1* and sometimes also *rrnL* transcripts, and its consensus sequence has been carefully characterized here, for the first time, in the “tick” group.

## Methods

### *I. ricinus* mtDNA Annotation and Analyses

The amplification and sequencing of the complete mtDNA of *I. ricinus* is described in Text S1. The mt sequence was deposited at EMBL database under accession number JN248424.

Protein coding genes (PCG) of *I. ricinus* were annotated by sequence similarity to the orthologous PCGs of other ticks. Partial stop codons were assumed only to avoid overlap with a downstream gene located on the same strand, while the 3′-end of *nad1* was experimentally identified by 3′ RACE and EST analyses (see below). Overlaps between genes located on the same strand were kept as short as possible. tRNA annotation was performed comparing the predictions of tRNAscan-SE [29] and ARWEN [30] to the tRNAs annotated in other ticks (LocARNA multi-alignment [31]). Small (*rrnS*) and large (*rrnL*) ribosomal subunit rRNAs were identified by sequence similarity and their boundaries were settled as adjacent to those of the flanking genes. As an exception, the 3′-end of *rrnL* was experimentally determined by 3′ RACE and EST analyses (see below).

In the *I. ricinus* mtDNA analyses, the gene boundaries of the 10 previously published mtDNAs of Ixodida (Table 1) were revised based on sequence multi-alignment, transcriptional data, and the criterion of “minimum gene overlap”. Using this approach, we optimized the annotation of a total of 93 genes (i.e., 60 tRNAs and 33 PCGs) in 10 species, with up to 14 gene boundaries modified in *I. hexagonus* (data available on request).

**Table 1:** Completely sequenced mitochondrial genomes of Ixodida.

Family	Group	Subfamily	Species	mtDNA
Ixodidae	Prostriata	Ixodinae	*Ixodes ricinus*	This study
“	“	“	*Ixodes hexagonus*	NC_002010
“	“	“	*Ixodes persulcatus*	NC_004370
“	Australasian Prostriata	“	*Ixodes holocyclus*	NC_005293
“	“	“	*Ixodes uriae*	NC_006078
“	Metastriata	Amblyomminae	*Amblyomma triguttatum*	NC_005963
“	“	Haemaphysalinae	*Haemaphysalis flava*	NC_005292
“	“	Rhipicephalinae	*Rhipicephalus sanguineus*	NC_002074
Argasidae		Ornithodorinae	*Carios capensis*	NC_005291
“		“	*Ornithodoros moubata*	NC_004357
“		“	*Ornithodoros porcinus*	NC_005820

Secondary structures of the major non-coding region (the control region; CR) were predicted with Mfold [32].

Exact direct repeats longer than 9 bp were searched in the mtDNA sequences with RepFind [33], setting the P-value cut-off at 0.01 and with no filter for low-complexity sequences.

The Tick-Box motif was searched in complete and partial mt sequences using PatSearch [34], [35]. The Tick-Box consensus sequence (ttgyrtchwwwtwwgda) was defined as the sequence with the highest sensitivity in PatSearch analyses against all analysed Ixodida species. Tick-Box searches in the whole mtDNA sequences of two Xiphosura and 14 *Drosophila* species were carried out allowing mismatches and/or indels to the original consensus sequence. Tick-Box sequence logos [36] were generated by WebLogo [37] using all occurrences of the Tick-Box in the analysed species (Table S1). The possible presence of conserved secondary structure around the Tick-Box was verified by LocARNA [31].

Gene order, non-coding regions, and gene sequences of all mtDNAs analysed in this study were retrieved from MitoZoa Rel. 9.1 [38], [39] (http://www.caspur.it/mitozoa), a database collecting one representative and manually-curated mtDNA entry for each metazoan species. Therefore, the 474 complete *nad1* sequences of arthropods analysed in this study were retrieved from MitoZoa Rel. 9.1.

### 3′ RACE of *rrnL* and *nad1* Genes

Since both *rrnL* and *nad1* transcripts are polyadenylated in *Drosophila melanogaster* [26], [27], [40], the 3′-end of these transcripts was identified by 3′ RACE (Random Amplification of cDNA End) or by identification of the start site of the polyA tail in mitochondrial ESTs, according to the method used in [25]. The 3′ RACE of *nad1* and *rrnL* transcripts of *I. ricinus* was carried out using gene-specific inner (nad1-620pr and rrnL-1050pr) and outer (nad1-173pr and rrnL-850pr) primers (see Text S1).

One partially engorged adult female of *I. ricinus* was collected in Monte Bollettone (Como, Italy) and the total RNA was extracted following the total RNA isolation procedure of the mirVana™ miRNA Isolation Kit (Ambion). RNA was retrotranscribed to cDNA using an adaptor-ligated oligo (dT)-primer (FirstChoice RML-RACE Kit, Invitrogen) and the reverse transcriptase of the QuantiTect Reverse Transcription Kit (Qiagen). The first PCR reaction was assembled coupling the 3′ RACE outer adaptor primer (FirstChoice RML-RACE Kit, Invitrogen) with the nad1-173pr or rrnL-850pr primer. The second nested PCR reaction was assembled coupling the 3′ RACE inner adaptor primer (FirstChoice RML-RACE Kit, Invitrogen) with the nad1-620pr or rrnL-1050pr primer. All PCR reactions were performed in a total volume of 25 µl with 1.25 units of GoTaq (Promega), according to the manufacturer’s protocol. A single band of approximately the expected size was observed as product of the inner and outer PCRs in both the *nad1* and *rrnL* 3′ RACE. In order to identify possible alternative polyadenylation sites located few nucleotides apart, nested PCR products were cloned (CloneJET PCR Cloning Kit, Fermentas) and a total of six positive clones were sequenced for each fragment. The partial RNA sequences of the *I. ricinus rrnL* and *nad1* transcripts were deposited at EMBL database under accession numbers HE798553, HE798554 and HE798555.

### EST Analyses of *rrnL* and *nad1* Genes

EST sequences highly similar to the *rrnL* and *nad1* genes of a given tick species were identified by Blast search [41] using as a probe the mt gene sequence of the same or of a congeneric species. Blast searches were carried out against the “Est_other” database that, at February 2012, included 297,856 ESTs of 20 Ixodida species. ESTs with statistically significant matches were assembled together with the corresponding mitogenomic sequence using Geneious [42]. The polyA start site was identified by visual inspection of the assembly. In particular, “A” or “T” stretches >10 bp located at the end of EST sequences were considered equivalent to the polyA tail of a mature transcript. In some cases, the lack of EST quality data and/or the presence of A stretches on the genomic mtDNA does not allowed mapping this site with single-nucleotide resolution, but only in a range of 2–5 nucleotides. The *rrnL* polyA site of *Boophilus microplus* and *Dermacentor andersoni,* and the *nad1* polyA site of *L. polyphemus* were determined by analysis of the original untrimmed ESTs, kindly provided by the authors (see Acknowledgements).

### Phylogenetic Analyses

Phylogenetic analyses were performed on the 13 PCGs of the 10 complete mtDNA of Ixodida (Table 1), using Argasidae as outgroup species. PCGs were aligned at the amino acid level with Muscle [43], and the equivalent nucleotide alignments were generated by “back-translation”. Ambiguous alignment regions were trimmed with Gblocks [44] using default parameters. The single PCG alignments were then concatenated with SEAVIEW [45].

Bayesian phylogenetic analyses were carried out on both amino acid and nucleotide alignments. The evolutionary models best fitting to the analyzed datasets were selected with ProtTest 1.4 [46] for amino acid, and ModelTest [47] for nucleotide datasets, according to the Akaike Information Criterion (AIC). The selected substitution model was the MtArt [48] with a proportion of invariant sites (I) and a gamma distribution for rate heterogeneity across sites (Γ) for the amino acid dataset, and the GTR+I+Γ for the nucleotide dataset [49]. Bayesian trees were calculated using MrBayes 3.1.2 [50]. Due to the absence of MtArt, the more general GTR and MtRev [51] model were applied in the amino acid analyses. Two different partitions based on the 13 genes and on the 3 codon positions were used in the nucleotide dataset analysis. One partition based on the 13 different proteins was used for the amino acid dataset. Two parallel analyses, each composed of one cold and three incrementally heated chains, were run for 2.5 million generations. Trees were sampled every 100 generations and burn-in fraction was calculated as 25% of total sampled trees, according to lnL stationary analyses.

## Results and Discussion

### *Ixodes ricinus* Genome Organization and Phylogeny

The mtDNA of *I. ricinus* is 14,566 bp long and encodes the 37 mt genes typical of other metazoans. The general features of this genome, together with peculiarities of the protein-coding genes (PCGs), the tRNA genes, the control region, and the small non-coding regions (NCRs) are illustrated in the Text S1, Figure S1 and Figure S2.

**Figure 1:**
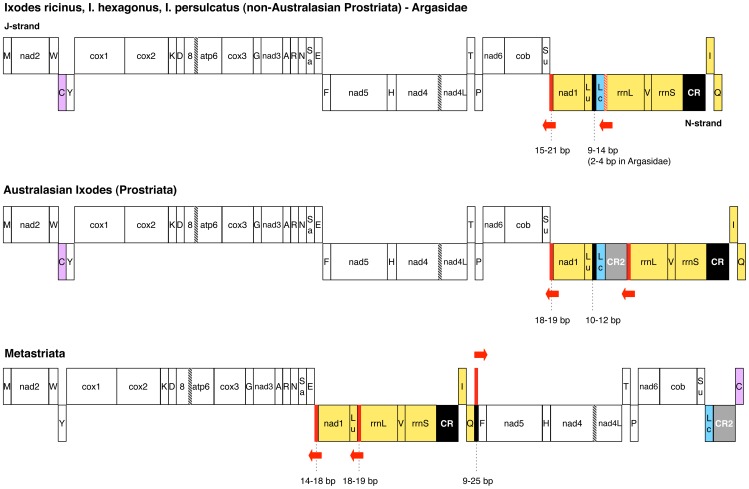
Mitochondrial gene arrangement of *Ixodes ricinus* and 10 other Ixodida species. Translocated genes are reported in the same colour. Black block: non-coding regions ≥9 bp in all species of a taxonomic group, with bp range indicated by dashed lines; red block: Tick-Box within a non-coding region; red-hatched block: Tick-Box overlapped to *trnL(CUN)*; red arrow: direction of the Tick-Box; black-hatched block: overlaps between genes; grey block: duplicated control region. The majority (J) and the minority (N) DNA strands, defined by the number of encoded genes, are also indicated. Gene abbreviations: 8, atp6: subunits 8 and 6 of the F0 ATPase; cox1-3: cytochrome c oxidase subunits 1–3; cob: cytochrome b; nad1–6 and nad4L: NADH dehydrogenase subunits 1–6 and 4L; rrnS and rrnL: small and large subunit rRNAs. tRNA genes are indicated by the one-letter code of the transported amino acid, with Lu: *trnL(UUR);* Lc: *trnL(CUN);* Sa: *trnS(AGN)*; Su: *trnS(UCN)*. Analysed mtDNAs are listed in Table 1.

**Figure 2:**
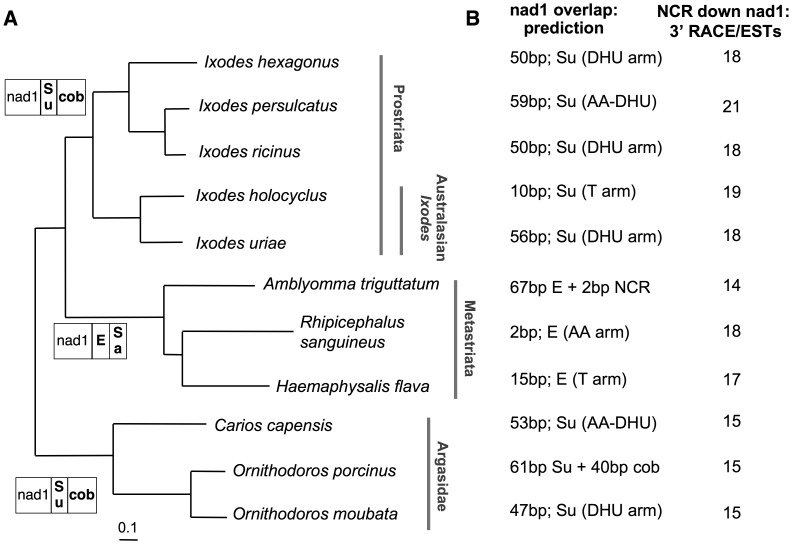
Features of the *nad1* 3′-end and the downstream non-coding region, mapped on the Ixodida phylogeny. (A) Ixodida Bayesian tree calculated on the nucleotide sequence of the 13 mt protein-coding genes, and gene order downstream of *nad1*. Bayesian tree was calculated according to the GTR+I+gamma model, using 13 partitions, and all branches have a posterior probability value equal to 1. In gene order scheme, the genes encoded by the strand opposite to that of *nad1* are reported in bold. Gene abbreviations as in Figure 1. (B) Predicted overlap between *nad1* and the downstream gene, and length of the non-coding region experimentally identified downstream of *nad1* by transcriptional data. tRNA regions containing the *nad1* complete stop codon are indicated in brackets, with the following abbreviations: DHU: DHU arm; AA: amino acid acceptor arm; AA-DHU: spacer between the AA and DHU arms. Gene abbreviations as in Figure 1.

Figure 1 compares the genome organization of all available complete mtDNAs of ticks, taking also into account the location of the control region (CR), which contains the regulatory elements of mt transcription and replication. The genome organization of *I. ricinus* is identical to that found in all other available non-Australasian *Ixodes* species and in Argasidae (Figure 1). Since it is also shared with *L. polyphemus,* this organization is considered to be ancestral to all arthropods [10], [11], [52]. Australasian *Ixodes* species (*I. uriae* and *I. holocyclus*) have a genome organization almost identical to that of other *Ixodes* and Argasidae, except for the presence of a duplicate control region (CR2) between *trnL(CUN)* and *rrnL* (Figure 1), suggesting possible differences in mtDNA replication/transcription mechanisms [23]. With respect to the *I. ricinus* genome organization, the Metastriata exhibit: (1) the translocation of a large genomic block comprising 7 genes and the CR (yellow block in Figure 1); (2) the translocation plus inversion of *trnC* (violet blocks in Figure 1)*;* (3) the presence of a duplicate CR2 between *trnL(CUN)* and *trnC* (grey blocks in Figure 1) [24], [53]. As already observed, the duplicate CR2s of both Metastriata and Australasian *Ixodes* exhibit concerted evolution and probably originated, together with the identified genome rearrangements, through two distinct events of tandem duplication and random gene loss [23], [53].

All Bayesian phylogenetic analyses of Ixodida, carried out on the 13 PCGs at both nucleotide and amino acid level, give congruent results and support the monophyly of the major Ixodida lineages. In particular, all phylogenetic reconstructions unambiguously identifies *I. ricinus* as sister taxon to *I. persulcatus,* with non-Australasian *Ixodes* positioned in a distinct highly supported clade (see nucleotide Bayesian tree in Figure 2A). This topology is in agreement with previous phylogenies based only on molecular data [2], [3], [54] or based on both morphological characters and nucleotide sequences (18S and 28S nuclear rRNAs; 16S mt rRNA) [55].

### Partial Stop Codons and *nad1* Annotation

In the mtDNA, partial stop codons are completed by polyadenylation of mature transcripts that are produced by endonucleolytic cleavages of precursor RNAs at sites immediately adjacent to tRNA genes [26], [27], [28]. It should be also noted that the usage of a partial stop codon eliminates the overlap between two consecutive genes (a PCG and a tRNA) encoded by the same strand, allowing the production of two full-length transcripts by cleavage of the same polycistronic RNA precursor. Thus, partial stop codons are commonly predicted according to the presence of an abutted tRNA gene and to the rule of “minimum overlap” between genes encoded by the same strand. In *I. ricinus,* the partial stop codons of five PCGs can be predicted according to the above-described rules (partial “T” stop codon in *cox2*, *cox3*, *nad5,* and *cob;* “TA” in *nad2*). On the contrary, the identification of the correct stop codon of *nad1* is quite tricky because the 3′ end of this gene has unique peculiarities that do not fit to the known transcript maturation process and the derived annotation rules. In particular, *nad1* is the only PCG followed by a gene encoded on the opposite strand (Figure 1). Therefore, based on the punctuation model of transcript maturation, the annotation of a partial stop codon is not strictly required in this case, since *nad1* and the downstream gene are transcribed by two different strands. Moreover, the complete stop codon of the *nad1* ORF is surprisingly located well inside the opposite strand-encoded *trnS(UCN)* gene, producing a large gene overlap of 50 bp.

Strikingly, even the 3′-ends of the *nad1* genes/proteins currently annotated in all other ticks present similar unusual features.

Firstly, in almost all published tick mtDNA [23], [24], [53], the currently annotated 3′-end of *nad1* has a complete stop codon located inside the first or even the second downstream, opposite strand-encoded, gene. This annotation gives rise to a gene overlap whose size is highly variable between species and ranges from 2 to 101 bp (Figure 2B). The most extreme case is in the argasid tick *Ornithodoros porcinus*, where the annotated *nad1* contains the reverse complement of the entire downstream *trnS(UCN)*, and the complete *nad1* stop codon is located inside the following *cob* gene. Similarly, the *nad1* ORF of the metastriate *A. triguttatum* contains the entire *trnE* gene. It is noteworthy that the predicted *nad1* overlap size is not related to species phylogeny or gene order around *nad1* (Figure 2), and that the currently annotated *nad1* complete stop codons fall in different regions of the downstream tRNA gene, depending on the species (Figure 2B).

Secondly, assuming the veracity of these complete stop codons, the *nad1* protein of Ixodida should have an extra C-terminal tail compared to the *nad1* of *D. melanogaster*, ranging from 6 to 38 amino acids (20 amino acids in *I. ricinus*). The analysis of a multi-alignment of 474 *nad1* proteins belonging to different arthropod species (see Materials and Methods) shows that this putative C-terminal tail is Ixodida-specific, being absent in all other available chelicerates (45 species) and in 96% of the whole arthropod dataset. Finally, this putative C-terminal tail has a low amino acid sequence similarity even within Ixodida (data not shown).

All these peculiarities prompted us to experimentally determine the actual *nad1* stop codon of Ixodida by: (a) 3′ RACE in *I. ricinus*; (b) identification of the polyA start site of *nad1* ESTs in all other tick species for which EST data are available.

**Figure 3:**
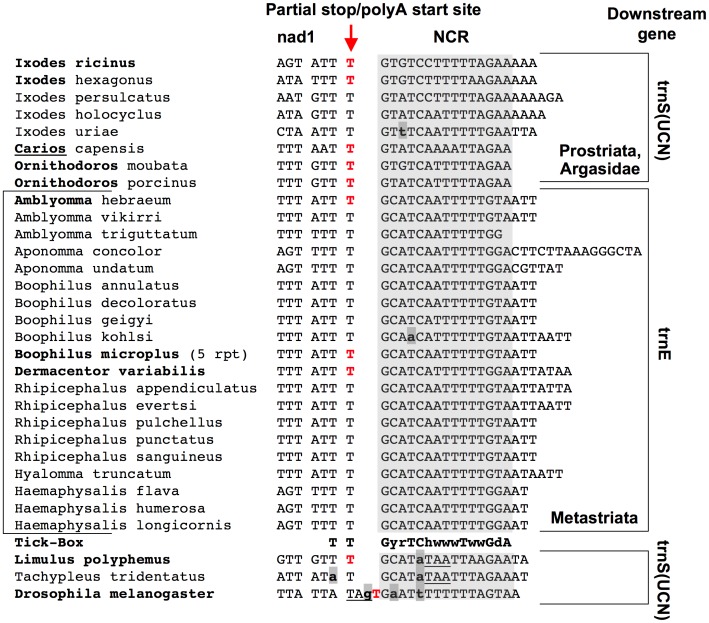
Non-coding region between *nad1* and *trnS(UCN)/trnE,* and the Tick-Box degenerate consensus sequence. Bold face: species listed in Table 2, for which the 3′-end of the *nad1* transcript was experimentally determined by ESTs or 3′ RACE. Bold face only for the genus name: when the DNA sequence of a given species was unknown, the 3′-end of *nad1* reconstructed by ESTs was mapped on the sequence of a congeneric species. Bold and underlined genus name: the *nad1* 3′-end of the argasid *Argas monolakensis* (Table 2) was mapped on the sequence of the argasid *Carios capensis*. Red colour: last DNA-encoded nucleotide preceding the *nad1* polyA tail. Underlined nucleotides: complete stop codons predicted *in silico*; bold lower case nucleotides with grey background: differences to the Tick-Box consensus sequence; rpt: presence of a repeated sequence containing the Tick-Box (see main text). Degenerate nucleotide symbols according to the IUPAC code. Analyses species and sequence accession numbers are listed in Table S1. Gene abbreviations as in Figure 1.

In *I. ricinus*, the 3′ RACE analysis shows that the *nad1* mRNA ends with a TAA stop codon created by the polyA tail and located exactly at the same position of the complete DNA-encoded stop codon of *D. melanogaster* (Figure 3). *nad1* ESTs confirm this site in *I. ricinus* and in seven additional tick species (*Ixodes scapularis*, three Argasidae and three Metastriata species; see Table 2 and Figure 3). These data unambiguously demonstrate that, in all major Ixodida lineages, the putative C-terminal tail and the gene overlap of *nad1,* predicted *in silico,* result from the misannotation of the actual *nad1* stop codon. Most importantly, the accurate annotation of the *nad1* 3′-end by transcriptional data identifies an unexpected NCR between *nad1* and the downstream tRNA encoded by the opposite J-strand (i.e., *trnS(UCN)* in Argasidae and Prostriata, and *trnE* in Metastriata). This NCR has been identified in all the 11 analysed complete mtDNAs (Figure 2B) and in all the partial mt sequences available for other 17 tick species (Figure 3 and Table S1). We can thus conclude that the NCR downstream of *nad1* is a common and ancestral character of the Ixodida mtDNA.

**Table 2:** ESTs matching to the *nad1* gene, and *nad1* ESTs with a polyA stretch corresponding to the polyA tail of the mature *nad1* transcript.

Taxon	Species	AvailableESTs^a^	*nad1* ESTs		mtDNA^c^
			tot	polyA^b^	
Prostriata	*Ixodes ricinus*	1,969	2	2	This study
Prostriata	*Ixodes scapularis*	193,773	42	3	congeneric
Argasidae	*Ornithodoros coriaceus*	923	1	1	congeneric
Argasidae	*Ornithodoros parkeri*	1,563	1	1	congeneric
Argasidae	*Argas monolakensis*	2,914	7	6	–
Metastriata	*Amblyomma rotundatum*	1,230	2	1	congeneric
Metastriata	*Dermacentor variabilis*	2,090	8	7	AY059254s1
Metastriata	*Boophilus microplus*	52,901	12	4	AF110621
Xiphosura	*Limulus polyphemus*	8,488	12	7	NC_003057

a ESTs publicly available at 20 Feb, 2012.

b ESTs with a terminal polyA stretch >10 bp, mapping to the end of *nad1*. Polyadenylated ESTs mapping well inside the *nad1* gene have not been considered, as they are mostly cDNA artefacts originated from the annealing of the oligo-dT primer to an A-rich inner gene region during the cDNA first strand synthesis.

c accession number of the mt genomic sequence, if available. “Congeneric” means that only the sequence of a congeneric species is available, as reported in Table S1.

As shown in Figure 3, this NCR is AT-rich (mean AT% = 76%), ranges from 14 to 30 bp in length, and is characterized by the presence of a degenerate 17 bp motif that includes the two last conserved nucleotides of *nad1*. Moreover, it can be observed that:

1. this degenerate motif is associated with the 3′-end of *nad1* even when *nad1* is translocated in Metastriata (Figure 1);
2. this motif is located at the boundaries between two large blocks of genes encoded by opposing genomic strands (Figure 1);
3. the polyA start site of the *nad1* mRNA does not map at the boundary of the downstream tRNA gene in any analysed tick (Figure 3), thus excluding a *nad1* transcript maturation according to the tRNA punctuation model [27], [28];
4. this motif is absent in the *nad1* mature transcript, thus its sequence is either un-transcribed or quickly removed from the *nad1* precursor transcript.

All these data suggest that this motif, that we have named the “Tick-Box”, directs the 3′-end formation of the polyadenylated *nad1* transcripts in Ixodida, and likely works as a maturation signal for the cleavage of a large precursor transcript, or as a transcription termination signal.

We need to stress that this motif has been originally included inside the *nad1* gene, and its identification has been made possible starting from the observations of: i) unusual position of the complete stop codon of *nad1*; ii) unusually large overlap between genes encoded by opposite strand; iii) an extra not-conserved C-terminal tail in the *nad1* proteins of ticks. Thus, far from being a simple case of *nad1* misannotation, this is an emblematic case that emphasizes how detailed analyses of unusual gene features can help to identify hidden functional element, and how gene misannotations can hamper the recognition of conserved regulatory elements.

### The Tick-Box Downstream of *rrnL*

Sequences similar to the Tick-Box motif were sought along the entire mt sequences of all 11 ticks (Table 1) using pattern matching software, and were found to be present in only two or three fixed genomic positions (red blocks in Figure 1):

1. downstream of *nad1*;
2. near the 3′-end of *rrnL*;
3. inside a small NCR located between *trnQ* and *trnF* in some Metastriata.

Available partial mt sequences of 41 additional prostriates and metastriates (Table S1) contain Tick-Box motifs only in these genomic positions.

The exact location of Tick-Box motif near the 3′-end of *rrnL* depends on the taxa, indeed this Tick-Box falls:

1. in the DHU and anticodon arms of *trnL(CUN)* in Argasidae and non-Australasian *Ixodes* lineages (Figures 1);
2. at the end of CR2 in Australasian *Ixodes* (Figure 1)*;*
3. a few bp upstream of the 3′-end of the currently annotated *rrnL* in Metastriata (Figure 1).

In order to study the potential functional role of the *rrnL* associated Tick-Box, we experimentally determined the 3′-end of *rrnL* transcripts through 3′ RACE in *I. ricinus,* and by using EST data in 10 other species (Table 3).

**Table 3:** ESTs matching to the *rrnL* gene, and *rrnL* ESTs with a polyA stretch corresponding to the polyA tail of the mature *rrnL* transcript.

Taxon	Species	Available ESTs^a^	*rrnL* ESTs	polyA ESTs^b^	polyA start at:		mtDNA^c^
					5′-end of Tick-Box	Other	
Prostriata	*Ixodes ricinus*	1,969	23	13	13	0	This study
Prostriata	*Ixodes scapularis*	193,773	33	8	8	0	AB161439
Argasidae	*Ornithodoros coriaceus*	923	20	5	4	1	congeneric
Metastriata	*Amblyomma americanum*	6,480	852	30	30	0	congeneric
Metastriata	*Amblyomma rotundatum*	1,230	18	14	12	2	congeneric
Metastriata	*Amblyomma tuberculatum*	387	17	2	2	0	congeneric
Metastriata	*Hyalomma anatolicum*	736	5	5	2	3	congeneric
Metastriata	*Hyalomma marginatum*	2,110	27	24	18	6	congeneric
Metastriata	*Dermacentor andersoni*	1,387	67	25	16	9	congeneric
Metastriata	*Boophilus microplus*	52,901	296	130	115	15	AF110619
Metastriata	*Rhipicephalus sanguineus*	2,899	428	57	50	7	NC_002074
Xiphosura	*Limulus polyphemus*	8,488	2	2	0	2	NC_003057

a ESTs publicly available at 20 Feb, 2012.

b ESTs with a terminal polyA stretch >10 bp, mapping to the end of *rrnL*. Polyadenylated ESTs mapping well inside the *rrnL* gene have not been considered, as they are mostly cDNA artefacts originated from the annealing of the oligo-dT primer to an A-rich inner gene region during the cDNA first strand synthesis.

c accession number of the mt genomic sequence, if available. “Congeneric” means that only the sequence of a congeneric species is available, as reported in Table S1.

**Figure 4:**
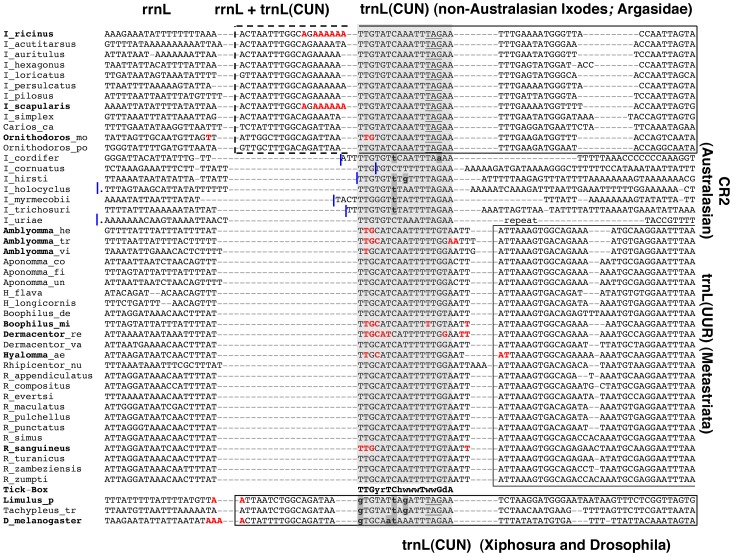
The Tick-Box motif located downstream of *rrnL*. Bold face: species listed in Table 3, for which the 3′-end of the *rrnL* transcript was experimentally determined by ESTs or 3′ RACE. Bold face only for the genus name: when the DNA sequence of a given species was unknown, the 3′-end of *nad1* reconstructed by ESTs was mapped on the sequence of a congeneric species. The *rrnL* 3′-end of *Hyalomma anatolicum* and *marginatum* (Table 3) were both mapped on the sequence of *Hyalomma aegyptium* (*Hyalomma_ae*). Red colour: last DNA-encoded nucleotide preceding the *rrnL* polyA tail. Dashed line: overlap between *rrnL* and *trnL(CUN)*. Genus names were abbreviated to a single letter for *Ixodes* (I), *Haemaphysalis* (H), *Rhipicephalus* (R) and *Drosophila* (D). Underlined nucleotides: tRNA anticodon; bold lower case nucleotide with grey background: differences to the Tick-Box consensus sequence; blue lines: original annotation of the *rrnL* 3′-end, with a dot indicating the presence of additional nucleotides; “repeat”: 71 bp-long inverted repeat located in the CR2 and *rrnL* gene of *I. uriae* (position 12431–12501 and 12606–12676, respectively, of NC_006078). Degenerate nucleotide symbols according to the IUPAC code. Analyses species and sequence accession numbers are listed in Table S1. Gene abbreviations as in Figure 1.

In *I. ricinus*, the *rrnL* polyadenylated transcript ends at two alternative sites, separated by 1 bp and located inside *trnL(CUN)*, immediately before the 5′-end of the Tick-Box motif (red sites in Figure 4). Indeed, most *rrnL* 3′ RACE clones stop 14–19 bp inside *trnL(CUN)*, while only one clone stops 11–12 bp inside *trnL(CUN)*: the presence of one/multiple “A” nucleotides on the mitogenomic sequence prevents precisely mapping these polyA start sites. Even *rrnL* ESTs of *I. ricinus* confirm these two alternative 3′-ends of *rrnL*. Moreover, these ESTs do not provide support for the existence of *rrnL* transcripts terminating at the 5′-end of *trnL(CUN)*, as predicted by the tRNA punctuation model.

In *I. scapularis,* EST data identify the 3′-end of *rrnL* at two sites corresponding exactly to those found in *I. ricinus* (Table 3 and red sites in Figure 4). In *Ornithodoros* (Argasidae) and in all analysed metastriates, *rrnL* terminates always at the beginning of the Tick-Box. Moreover, in *Ornithodoros* an additional *rrnL* 3′-end site is located at the 5′-end of *trnL(CUN),* and in some metastriate species additional *rrnL* 3′-end sites can be observed very close to the 5′-end of the nearby *trnL(UUR)* gene, as predicted by the tRNA punctuation model (Table 3, and red sites in Figure 4). However, in each analysed species the majority of ESTs support the positioning of the *rrnL* 3′-end at the beginning of the Tick-Box motif (Table 3), suggesting that this site could be used more frequently than the other (given the different nature of the original cDNA libraries, definitive quantitative data cannot be inferred. Moreover, in some species the lack of EST quality data and/or of the mitogenomic sequence does not allow mapping of the *rrnL* polyA start site at single-nucleotide resolution). The lack of ESTs for Australasian *Ixodes* precludes validation of the 3′-end of *rrnL* in this lineage. However, based on sequence similarity to other Prostriata and on the lack of a tRNA abutted to *rrnL*, we hypothesize that in Australasian *Ixodes* species the 3′-end of *rrnL* occurs immediately before the identified Tick-Box motif (Figure 4).

In conclusion, as for *nad1*, transcriptional data are consistent with a functional role of the Tick-Box sequence in the 3′-end formation of polyadenylated *rrnL* transcripts. Indeed, in all analysed species the *rrnL* polyA tail starts immediately before or within the first 5 nt of the Tick-Box motif, independently of the gene/NCR downstream of *rrnL*. All additional *rrnL* polyadenylation sites, observed mainly in Metastriata, conform to the predictions of the tRNA punctuation model (i.e., they fall at the 5′-end of the downstream tRNA gene, considering the ambiguities due to EST quality) and appear infrequently used, as roughly estimated by the number of supporting ESTs (Table 3).

The presence of a Tick-Box near the 3′-end of *rrnL* is intriguing since a transcription termination signal has been functionally identified downstream of *rrnL* in Mammalia: this signal is a tridecamer sequence entirely contained in the *trnL(UUR)* gene [56], [57] and functions as a binding site for the mitochondrial transcription termination factor (mTERF) [58], [59]. Based only on sequence similarity to this mammalian tridecamer sequence, Valverde et al. [60] identified a “TGGCAGA” heptamer conserved downstream of *rrnL* from mammals to insects and protozoans, and hypothesized its function as an “rRNA termination box”. However, later functional studies have not validated the Valverde’s “rRNA termination box” as a binding site to the mTERF homologs of sea urchin and *D. melanogaster* [61], [62], [63], [64]. We need to stress that our Tick-Box does not coincide with the Valverde’s rRNA termination box either in sequence or exact genomic position. Moreover, unlike the rRNA termination box, our motif has been defined using both sequence similarity and transcriptional data. Finally, it should be noted that in Argasidae and non-Australasian *Ixodes* the exact location of the *rrnL* Tick-Box generates an overlap between *rrnL* and *trnL(CUN)* (dashed line in Figure 4). This situation recalls the overlap between *rrnL* and *trnL(UUR)* found in mammals because of the presence of the *rrnL* transcription termination signal inside *trnL(UUR)* [65].

As in the case of *nad1*, the determination of the *rrnL* 3′-end by transcriptional data has allowed the discovery of: i) an unexpected NCR downstream of *rrnL* in Metastriata (11–22 bp long); ii) an overlap between *rrnL* and *trnL(CUN)* in Argasidae and non-Australasian *Ixodes* (12–19 bp long; see dashed line in Figure 4); iii) the misannotations of *rrnL* in most ticks (Figure 4). However, we need to emphasize that the determination of the exact boundaries of *rrnL* only by comparative analyses is complicated by difficulties in the prediction of the rRNA secondary structure and by the low sequence conservation at both ends of this gene.

### The Tick-Boxes of Metastriata

**Figure 5:**
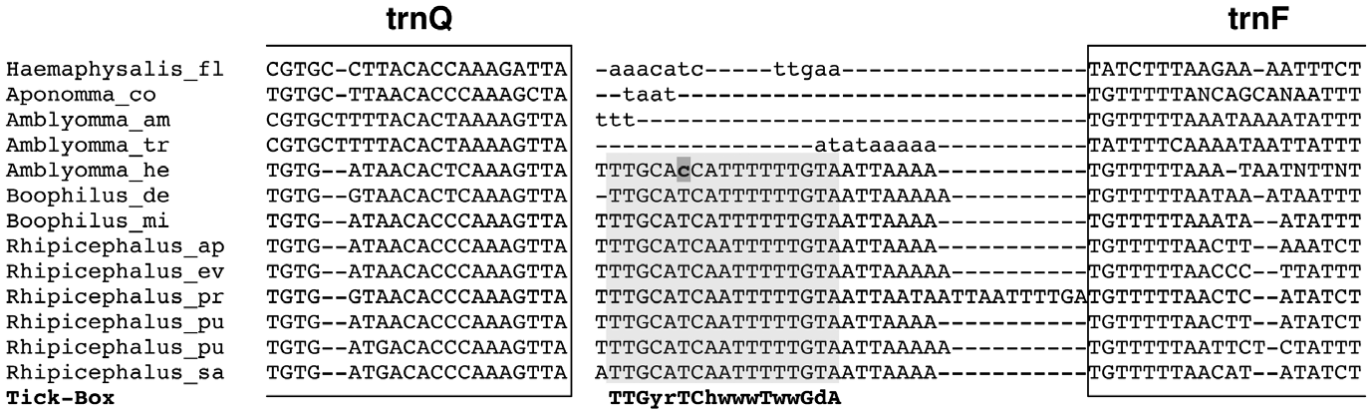
Tick-Box motif located in the NCR between *trnQ* and *trnF* of Metastriata. Bold lower case with grey background: differences to the Tick-Box consensus sequence. Analysed species and sequence accession numbers are listed in Table S1.

As shown in Figure 5, a third Tick-Box motif is located in the NCR between *trnQ* and *trnF* in 9 out of 13 analysed metastriates (complete and partial mtDNAs, see Table S1). In the remaining 4 metastriates, the *trnQ*-*trnF* NCR is always shorter than 12 bp, and does not contain an even partial Tick-Box sequence.

This third Tick-Box is characterized by several oddities:

1. It is always on the opposite strand compared to the two Tick-Boxes situated downstream of *nad1* and *rrnL* in the same genome (red arrows in Figure 1);
2. It is located in a NCR shared only by Metastriata, since the *trnQ*-*trnF* gene adjacency is specific of the metastriate gene rearrangement. Thus, if present, this third Tick-Box sequence gives rise to an inverted repeat (21 bp-long) that flanks the large translocated mt region of Metastriata ranging from *nad1* to *trnQ* (yellow block in Metastriata of Figure 1). Even more surprisingly, in *B. microplus* [66] this large translocated mt region is preceded by a fivefold tandem repeat (126 bp unit) composed of *trnE*+Tick-Box+3′-end of *nad1,* and is followed by a single inverted copy of the Tick-Box sequence;
3. The phylogenetic distribution of this third Tick-Box is quite erratic, since it is absent in Haemaphysalinae, present in Rhipicephalinae, and present/absent even in congeneric species of Amblyomminae (Figure 5, and Table S1). Thus, it is difficult to discriminate between ancient or recent origins of this third Tick-Box.

As further peculiarity, the Tick-Box sequences present in the same mtDNA of metastriates are almost identical (maximum of 2 nt differences, observed only in one among the 13 analysed species), while the Tick-Boxes present in the same mtDNA of Argasidae and Prostriata species differ for 3–6 nucleotides. More interestingly, in the three complete mtDNAs of metastriates, the Tick-Boxes downstream of *nad1* and *rrnL* are located inside a perfect direct repeat of 28–30 bp. On the contrary, perfect direct repeats of the same size are absent in Argasidae and Prostriata. These data suggest that the Metastriata Tick-Box motifs likely undergo to concerted evolution, as the duplicated CR2 of these taxa [24], [53]. It should be noted that this observation does not hold for Australasian *Ixodes*, where the intra-genome Tick-Boxes differ for 4–5 nucleotides and the identified duplicated CR2s also evolve by concerted evolution [23]. Although we have no convincing explanations for this observation, we hypothesize that the strong intra-genome Tick-Box conservation in Metastriata is related to the peculiar mt gene arrangement of this taxon.

The functional role of this third Tick-Box is enigmatic, and the absence of EST data for the *trnQ*-*trnF* region complicates the verification of possible functional hypotheses. However, since the sequence of this third Tick-Box is almost identical to that of functional Tick-Boxes identified in the same genome, we suggest that even the third Tick-Box is functional. We could tentatively hypothesize that this third Tick-Box motif plays the role of terminating the transcription of the J-strand, started at the CR, downstream of *trnI*. Indeed, in metastriates the movement of *trnI* far away from the cluster of other J-encoded genes makes J-strand transcription after *trnI* pointless (compare the J-strand gene distribution of Prostriata/Argasidae to Metastriata in Figure 1). Such a role in the rearranged mtDNAs might have represented a selective constraint for the conservation of the third Tick-Box in Metastriata. Finally, the presence of the Tick-Box motif at both ends of the large translocated mt regions of Metastriata (yellow blocks in Figure 1) might suggest its involvement in recombination events responsible for genome rearrangements. Indeed, signs of recombination have been found in several chelicerates based on the observation of concerted evolution, gene conversion, and translocation of genes to the opposite strand [17], [20], [23], [67].

### Origin and Evolution of Tick-Box

**Figure 6:**
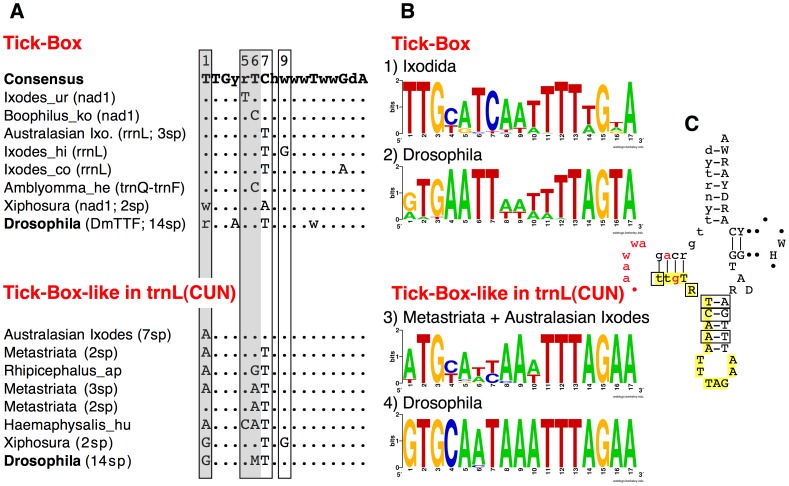
Tick-Box and Tick-Box-like sequences inside *trnL(CUN)* of Chelicerata and *Drosophila* species. (A) Consensus and variants Tick-Box motifs of Ixodida, Xiphosura, and *Drosophila* species, together with non-functional Tick-Box-like sequences overlapping *trnL(CUN).* Boxes: positions with nucleotide differences between Tick-Box and Tick-Box-like sequences; grey background: crucial positions discriminating functional Tick-Box from non-functional Tick-Box-like sequences (see main text). In brackets is reported the genomic position of the sequence (i.e., downstream *nad1*, downstream *rrnL*, or between *trnQ-trnF*) and the number of species (sp) showing that sequence. DmTTF: consensus binding sites of DmTTF in *Drosophila*, considering sequences located downstream *nad1* and between *trnE-trnF.* Analysed species and sequences are listed in Table S1. (B) Sequence logo for: (1) Tick-Box sequences of Ixodida; (2) DmTTF binding site of 14 *Drosophila* species, including both the sequences downstream of *nad1* and between *trnE*-*trnF*; (3) Tick-Box-like sequences inside *trnL(CUN)* of Metastriata and Australasian *Ixodes*; (4) Tick-Box-like sequences inside *trnL(CUN)* of 14 *Drosophila* species. Sequence logos were generated as described in Materials and Methods, using sequences listed in Table S1. (C) Consensus sequence and secondary structure of the *trnL(CUN)* genes of Argasidae and non-Australasian *Ixodes* containing a functional Tick-Box. Boxes: positions with nucleotide differences between Tick-Box and Tick-Box-like sequences; yellow background: Tick-Box motif; red colour: polyA starts sites determined by 3′ RACE or ESTs in Argasidae and non-Australasian *Ixodes*; lower case: overlap region between *rrnL* and *trnL(CUN)*; dot symbol: indels. Degenerate nucleotide symbols according to the IUPAC code. Analysed sequences are listed in Table S1.

Figure 6A shows the consensus sequence of the Tick-Box motif and few rare variants, differing only in 1 or 2 positions. Noteworthy, the Tick-Box consensus sequence is quite degenerate, showing nucleotide ambiguity codes in almost half of the 17 sites (Figure 6A). This relatively high degeneration of the Tick-Box consensus is in accordance with its nature of regulatory element, and can be related to its possible functioning through interactions with one or more nuclear-encoded proteins. Thus, as usual for regulatory elements, the precise sequence of the Tick-Box is quite different from one species to the other, and we expect this element to be subject to a taxon-specific evolution. In this respect, the Tick-Box shows an evolutionary pattern very similar to the CR, a mt region also known to evolve in a taxon-specific way [68]. Remarkably, in addition to the control region, the Tick-Box is the only NCR conserved in all Ixodida species (Figure 1), while all other NCRs of ticks are unalignable (even those located at the same relative position in different species) and mainly shorter than 9 bp (see Text S1).

The analysis of the single Tick-Box motifs indicates that the Tick-Box does not form a secondary structure, neither alone nor including surrounding sequences. The only exceptions are the few Tick-Boxes located downstream *rrnL* in non-Autralasian *Ixodes* and Argasidae, that are characterized by the overlapping with *trnL(CUN)* (Figure 6C). In these cases, the identified secondary structure has been evolutionary preserved because of the functional constraints of the tRNA gene rather than of the presence of the Tick-Box regulatory element (see also below). Therefore, the Tick-Box appears very different from the other hypothesized mt transcript processing sites. Indeed, in some cases, the absence of a tRNA punctuation mark has been supposed to be compensated by stem-loop structures resembling a tRNA portion [28].

In order to define the evolutionary origin of the Tick-Box, we have carefully investigated the presence of the Tick-Box in the basal chelicerate Xiphosura and in *Drosophila*, a highly derived insect genus belonging to the relatively recent Diptera lineage (divergence 228–245 Mya [69], [70]). These taxa have been selected due to their peculiar phylogenetic position and also because of the availability of a large amount of ESTs, useful for mt transcripts analyses. Moreover, there are several functional studies on the mt transcription of *D. melanogaster* [26], [27], [40], [64], [71], and the complete mtDNA is available for 14 congeneric *Drosophila* species (Table S1).

As for Xiphosura, we have considered the horseshoe crabs *L. polyphemus* (for which mtDNA and ESTs are available) and *Tachypleus tridentatus* (for which only the mtDNA is available). Our Xiphosura analyses show that:

1. Both species have a Tick-Box sequence (not perfectly matching to the Ixodida consensus) near the 3′-end of *nad1*. This Tick-Box includes the predicted *nad1* complete stop codon and a short downstream NCR (Figure 3). The ESTs of *L. polyphemus* show that the mRNA of *nad1* terminates immediately upstream of the Tick-Box sequence with a partial stop codon located at the same position of that of Ixodida (Table 2 and Figure 3). Thus, in Xiphosura the existence of a functional Tick-Box motif downstream of *nad1* is supported by both transcriptional and sequence data.
2. A divergent Tick-Box sequence (3 mismatches compared to the Ixodida consensus) can be identified near the 3′-end of *rrnL*, exactly inside *trnL(CUN),* in both horseshoe crabs (Figure 4). However, ESTs of *L. polyphemus* show that the 3′-end of *rrnL* transcript is not located at the beginning of the Tick-Box sequence but just at the 5′-end of *trnL(CUN)*, i.e., at the site predicted by the tRNA punctuation model (Figure 4 and Table 3). In conclusion, in Xiphosura a functional Tick-Box motif is absent downstream of *rrnL*, and the similar sequence identified inside *trnL(CUN)* probably results from the functional constraints of *trnL(CUN)*.

**Figure 7:**
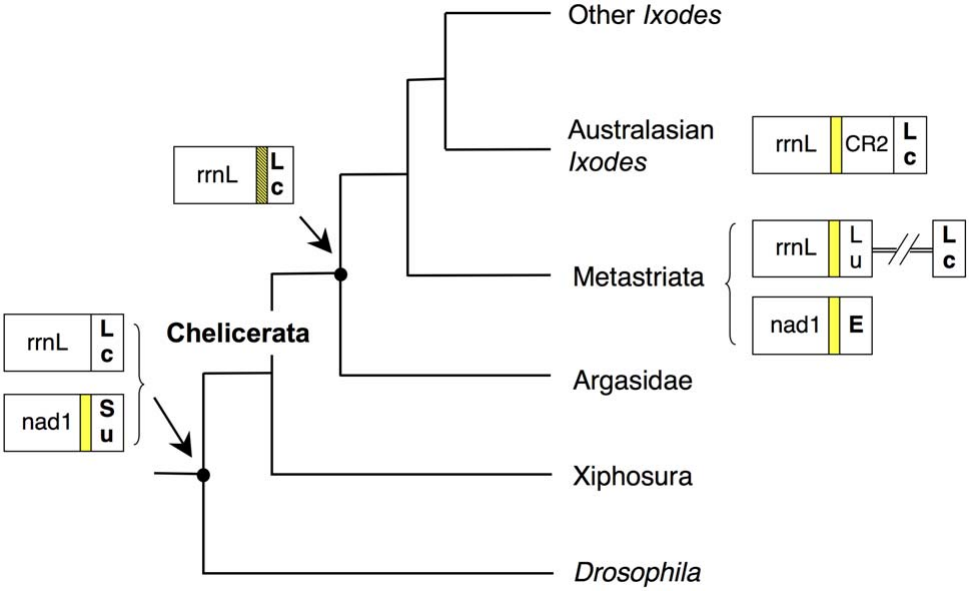
Evolutionary scenario of the Tick-Box motif in Ixodida and other arthropods. Tree topology according to [80]. Yellow block: Tick-Box motif; hatched yellow background: Tick-Box overlapped to *trnL(CUN)*; bold case: genes encoded by the J-strand.

Based on these data, we suggest that the Tick-Box downstream of *nad1* is an ancient signal that has been functionally conserved, in spite of the sequence changes, at least over the time separating Xiphosura from Ixodida (about 400 million years), while the Tick-Box downstream of *rrnL* is a specific invention of Ixodida (Figure 7). We hypothesize that the Tick-Box downstream of *rrnL* has evolved from a portion of *trnL(CUN)*, through acquisition of a new function related to post-transcriptional regulation (Figure 7). After this gain-of-function, the *trnL(CUN)* and the Tick-Box have become overlapped elements and have coevolved in Ixodida for long time, until genome rearrangement events have disrupted the adjacency *rrnL*-*trnL(CUN)* (two independent events: one in Metastriata and the other in Australasian *Ixodes*). We suggest that in these rearranged mtDNAs, the sequence including the two overlapped Tick-Box and *trnL(CUN)* elements has been duplicated, and then the two copies have started diverging. In particular, due to the need to regulate the *rrnL* 3′-end formation, the Tick-Box function has been preserved at the position immediately downstream of *rrnL*, where the *trnL(CUN)* function has been instead lost. On the contrary, in these rearranged mtDNAs, the Tick-Box function has been disrupted in the position actually preserving the *trnL(CUN)* function (Figure 7). Based on the proposed evolutionary scenario, the Tick-Box sequence downstream of *rrnL* in Metastriata and Australasian *Ixodes* should be the only remnant of a duplicated *trnL(CUN)*/Tick-Box sequence that has lost all but the essential *rrnL* post-transcriptional regulatory motif.

As for *Drosophila*, no sequence identical to the Tick-Box consensus motif is present in the whole mtDNA of *D. melanogaster* and congeneric species. However, the *D. melanogaster nad1* gene is followed by a 17 bp-long NCR that is one of the two binding sites of the DmTTF transcription termination factor, the other site being an almost identical sequence located between *trnE* and *trnF* [64], [71]. The consensus of the DmTTF binding site for the 14 available *Drosophila* species matches to the Tick-Box degenerate consensus of Ixodida in all but 3–4 positions (Figure 6A; logos n° 1 and n° 2 in Figure 6B). Noteworthy, the *D. melanogaster nad1* transcript is not 3′-processed at the site predicted by the tRNA punctuation model [27] but it terminates 16 bp upstream of the 5′-end of *trnS(UCN)* and 1 bp downstream the complete *nad1* stop codon (red colour in Figure 3). It should be also noted that *nad1* ends with a partial stop codon in 6 out of the 13 additional *Drosophila* mtDNAs, and that in all 14 available *Drosophila* species *nad1* is followed by a NCR ranging from 15 to 25 bp [72] and having a 41–65% identity to the *I. ricinus* NCR downstream of *nad1*. We conclude that the *Drosophila* has a Tick-Box signal downstream of *nad1* but this Tick-Box has a sequence quite divergent from the Ixodida consensus (Figure 7). This sequence variability between taxa follows the expected evolutionary pattern for a regulatory element, thus it is likely that the Tick-Box signal downstream of *nad1* is also present in other arthropod lineages with even more divergent sequences. We need also to emphasize that the Tick-Box of *Drosophila* functions as a binding site of DmTTF [64], [71].

In *D. melanogaster* the 3′-end of the *rrnL* polyadenylated transcript falls exactly at the site predicted by the tRNA punctuation model [27] (i.e., at the 5′-end of *trnL(CUN);* see Figure 4), and no DmTTF binding site is present immediately downstream of *rrnL* [64]. However, we identified a sequence similar to the Tick-Box inside the *trnL(CUN)* gene, which is located in all 14 *Drosophila* species just downstream of *rrnL*. This sequence shows 3 mismatches to the Tick-Box consensus sequence of Ixodida (Figure 4, Figure 6A, logo n° 4 of Figure 6B) and 76% identity to the *I. ricinus* Tick-Box inside *trnL(CUN).* As for horseshoe crabs, we conclude that in *Drosophila* there is no functional Tick-Box downstream of *rrnL* (Figure 7) and that the observed sequence conservation is due to the functional constraints of *trnL(CUN)*. It should be also noted that, in the comparison *D. melanogaster* - *I. ricinus*, the identity percentage is higher in the Tick-Box-like sequences overlapping *trnL(CUN)* than in the functional Tick-Boxes located in the NCR downstream *nad1* (76% and 65%, respectively). This indicates that the overlapping of Tick-Box to *trnL(CUN)*, and the degenerate nature of this Tick-Box regulatory signal can lead to misinterpretation of the Tick-Box presence/absence especially in taxa phylogenetically distant from Ixodida, and especially when only sequence similarity data are taken into account.

### Coevolution of Tick-Box and *trnL(CUN)*

To better investigate the coevolution of Tick-Box and *trnL(CUN)*, we have compared the identified Tick-Box motif to the similar sequences found inside the *trnL(CUN)* genes that lack a functional Tick-Box (Figure 6A). This comparison can assist in the identification of nucleotide positions discriminating functional Tick-Boxes from non-functional Tick-Box-like sequences overlapping *trnL(CUN).* The Tick-Box-like sequences overlapping *trnL(CUN)* were defined as non-functional based on EST data and mismatches to the Tick-Box consensus, and are present in the *trnL(CUN)* of Metastriata, Australasian *Ixodes,* Xiphosura, and all *Drosophila* species (see also Figures 3, 4). The logo of the Tick-Box-like sequences located inside *trnL(CUN)* is shown in Figure 6B, separately for ticks (logo n° 3) and *Drosophila* species (logo n° 4). Moreover, Figure 6C illustrates the consensus of functional Tick-Boxes located inside *trnL(CUN)* in non-Australasian *Ixodes* and Argasidae: as already discussed, these are the only Tick-Box elements showing a conserved secondary structure, since they are superimposed to a functional tRNA gene (see above).

As shown in Figure 6C (yellow background), the Tick-Box sequence inside *trnL(CUN)* is superimposed to half of the DHU and anticodon stems, plus the entire anticodon loop. According to the typical tRNA substitution pattern, the nucleotide substitutions observed in the *trnL(CUN)* with Tick-Box (boxed positions in Figure 6C) are mainly compensatory substitutions falling in the stem regions, while substitutions carefully avoid the anticodon loop. As reported in Figure 6A, the Tick-Box-like sequences inside *trnL(CUN)* differ in 2–4 positions from the consensus Tick-Box. As an exception, the Tick-Box-like sequence of Australasian *Ixodes* differs from the consensus Tick-Box only for a single substitution (T−>A) at position 1. Among positions with differences, we observe that positions 7 and 9 share the same substitution types in both Tick-Box variants and Tick-Box-like sequences (Figure 6A), thus these positions seem not to be crucial for the Tick-Box functionality. On the contrary, positions 5 and 6 (grey background in Figure 6A) have different substitution types in Tick-Box and Tick-Box-like sequences, indicating that they can be discriminating positions for the Tick-Box functionality. Finally, nucleotide substitutions at the first position of the consensus seem to inactivate the Tick-Box depending on the substitution type, the additional substitutions co-occurring in other positions, and the taxon (i.e., compare Xiphosura and *Drosophila* Tick-Box and Tick-Box-like sequences in Figure 6A). Thus, from the comparison between functional Tick-Boxes and Tick-Box like sequences, we can conclude that positions 1, 5 and 6 are the most important sites for the functionality of Tick-Box.

Overall, these data further support the hypothesis that Tick-Box is a highly dynamic and degenerate signal whose sequence variability is due to its specific regulatory function (i.e., the possible interaction with regulatory proteins encoded by the nuclear genome) and also to the overlap with coding sequences.

### Conclusions

In this study we describe the identification of the Tick-Box, a degenerate 17-bp DNA motif involved in post-transcriptional processes. In particular, Tick-Box directs the 3′-end formation of *nad1* and *rrnL* transcripts in all Ixodida lineages, as well as the 3′-end formation of the single *nad1* transcript in basal chelicerates of the Xiphosura order and in Diptera insects of the *Drosophila* genus. Although this motif is not restricted to tick species, it has been named “Tick-Box” because its consensus sequence has been here carefully characterized in Ixodida and because it is a “tick box” necessary for the 3′-end formation of some mt transcripts. We have not investigated in details the phylogenetic distribution of this motif in Chelicerata and Arthropoda, however its presence in *Drosophila* and *Limulus* suggest that it could be a quite ubiquitous signal, whose existence has been obscured by its taxon-specific evolutionary pattern and by its nature of post-transcriptional regulatory element. Indeed, as most regulatory elements, Tick-Box is a short and degenerate motif, showing a low sequence similarity within Ixodida and even lower sequence conservation in the more distant species of *Limulus* and *Drosophila*. Therefore, additional studies combining sequence similarity and transcriptional analyses are needed to define the Tick-Box consensus sequence in other arthropods and to clarify its phylogenetic distribution in the main arthropod groups.

With regard to the exact Tick-Box function, this element is associated to the 3′-end of the *nad1* and *rrnL* genes independently of the downstream gene/NCR. Moreover, it is absent in the mature transcripts. Therefore, we suggest that Tick-Box is either un-transcribed or quickly removed from the primary precursor transcripts of *nad1* and *rrnL*. According to this observation, Tick-Box might be one of the few exceptions to the tRNA punctuation model of mt transcript maturation [28] or a transcription termination signal, whose existence was originally hypothesized in *D. melanogaster* by Berthier [26]. Remarkably, the Tick-Box downstream of *nad1* found in *D. melanogaster* has been functionally described some time ago as one of the two binding sites of the DmTTF transcription termination factor [71]. Far from reducing the novelty of this study, the similarity between the DmTTF binding site of *D. melanogaster* and the Tick-Box downstream *nad1* of Ixodida supports the functional role of Tick-Box as a transcription termination site. Moreover, it testifies the poor link between functional and evolutionary studies on the mtDNA, and the difficulties of mere mt comparative analyses in the detection of regulatory elements. Indeed, to our knowledge, after its functional characterization, the binding site of DmTTF has not been further investigated at level of taxonomic distribution, consensus sequence or exact mitogenomic location(s) within the numerous available mtDNA sequences of arthropods.

The discrimination between the two hypothesized Tick-Box functions, precursor transcript maturation or transcription termination, can be experimentally tested in Prostriata and Metastriata by qualitative and quantitative analyses of the whole mt transcriptome and/or experiments aimed at demonstrating the binding of this motif by mt regulatory proteins, such as members of the MTERF protein family [73], [74]. The availability of cell lines for both these tick taxa can also help these analyses [75], [76].

Finally, we would like to emphasize that the small Tick-Box and the large mt control region are the only non-coding regions conserved in all mtDNAs of ticks. To our knowledge, there is only one other small NCR conserved in all mtDNAs of a large metazoan group, i.e. the L-strand replication origin (oriL) of vertebrates [77], [78]. The oriL is a 20–30 bp sequence embedded in a tRNA cluster and forms a stable stem-loop structure partially overlapped to *trnC*. On the contrary, the Tick-Box is a degenerate DNA motif that does not show a conserved secondary structure and, like the control region, is characterized by a taxon-specific evolution. Moreover, based on the presence of a third Tick-Box in Metastriata and of a second DmTTF binding site in *D. melanogaster,* we anticipate the presence of Tick-Box in different mitogenomic positions depending on the overall genome organization and on the details of the transcriptional process (i.e., number and type of transcriptional units).

## Supporting Information

Figure S1 **Putative secondary structure of the 22 tRNAs of** ***I. ricinus*** **.**
(PDF)

Figure S2 **Conserved motifs and secondary structures of the tick control region, mapped on the** ***I. ricinus*** **sequence.**
(PDF)

Table S1 **Accession numbers of 98 mitochondrial sequences belonging to 68 species analysed in this study.**
(XLS)

Text S1 **Amplification strategy and general features of tRNAs, control region, and small non-coding regions of the** ***I. ricinus*** **mtDNA.**
(DOC)

## References

[1] Dunlop JA, Selden PA (2009) Calibrating the chelicerate clock: a paleontological reply to Jeyaprakash and Hoy. Exp Appl Acarol 48: 183–197. 19199056 10.1007/s10493-009-9247-1

[2] Jeyaprakash A, Hoy MA (2009) First divergence time estimate of spiders, scorpions, mites and ticks (subphylum: Chelicerata) inferred from mitochondrial phylogeny. Exp Appl Acarol 47: 1–18. 18931924 10.1007/s10493-008-9203-5

[3] Mans BJ, de Klerk D, Pienaar R, Latif AA (2011) *Nuttalliella namaqua*: a living fossil and closest relative to the ancestral tick lineage: implications for the evolution of blood-feeding in ticks. PLoS One 6: e23675. 21858204 10.1371/journal.pone.0023675 PMC3157464

[4] Sonenshine DE (1991) Biology of ticks. Vol. 1. New York: Oxford Univ. Press. 447 p.

[5] Sonenshine DE (1993) Biology of ticks. Vol. 2 New York: Oxford Univ. Press. 465 p.

[6] Horak IG, Camicas JL, Keirans JE (2002) The Argasidae, Ixodidae and Nuttalliellidae (Acari: Ixodida): a world list of valid tick names. Exp Appl Acarol 28: 27–54. 14570115 10.1023/a:1025381712339

[7] Nava S, Guglielmone AA, Mangold AJ (2009) An overview of systematics and evolution of ticks. Front Biosci 14: 2857–2877. 10.2741/3418 19273240

[8] Keirans JE, Needham GR, Oliver JH (1999) The *Ixodes* (*Ixodes*) *ricinus* complex worldwide: Diagnosis of species in the complex, host and distribution. In: Glen R, Needham, Mitchell R, Horn DJ, Welbourn WC, editors. Acarology IX. Columbus, Ohio: The Ohio Biological Survey. 344.

[9] Sassera D, Beninati T, Bandi C, Bouman EA, Sacchi L, et al (2006) Candidatus *Midichloria mitochondrii,* an endosymbiont of the tick *Ixodes ricinus* with a unique intramitochondrial lifestyle. Int J Syst Evol Microbiol 56: 2535–2540. 17082386 10.1099/ijs.0.64386-0

[10] Boore JL, Collins TM, Stanton D, Daehler LL, Brown WM (1995) Deducing the pattern of arthropod phylogeny from mitochondrial DNA rearrangements. Nature 376: 163–165. 7603565 10.1038/376163a0

[11] Boore JL, Lavrov DV, Brown WM (1998) Gene translocation links insects and crustaceans. Nature 392: 667–668. 9565028 10.1038/33577

[12] Klimov PB, Oconnor BM (2009) Improved tRNA prediction in the American house dust mite reveals widespread occurrence of extremely short minimal tRNAs in acariform mites. BMC Genomics 10: 598. 20003349 10.1186/1471-2164-10-598 PMC2797822

[13] Masta SE (2000) Mitochondrial sequence evolution in spiders: intraspecific variation in tRNAs lacking the TPsiC Arm. Mol Biol Evol 17: 1091–1100. 10889222 10.1093/oxfordjournals.molbev.a026390

[14] Masta SE, Boore JL (2004) The complete mitochondrial genome sequence of the spider *Habronattus oregonensis* reveals rearranged and extremely truncated tRNAs. Mol Biol Evol 21: 893–902. 15014167 10.1093/molbev/msh096

[15] Masta SE, Boore JL (2008) Parallel evolution of truncated transfer RNA genes in arachnid mitochondrial genomes. Mol Biol Evol 25: 949–959. 18296699 10.1093/molbev/msn051

[16] Klimov PB, Knowles LL (2011) Repeated parallel evolution of minimal rRNAs revealed from detailed comparative analysis. J Hered 102: 283–293. 21422103 10.1093/jhered/esr005

[17] Masta SE (2010) Mitochondrial rRNA secondary structures and genome arrangements distinguish chelicerates: comparisons with a harvestman (Arachnida: Opiliones: *Phalangium opilio*). Gene 449: 9–21. 19800399 10.1016/j.gene.2009.09.009

[18] Park SJ, Lee YS, Hwang UW (2007) The complete mitochondrial genome of the sea spider *Achelia bituberculata* (Pycnogonida, Ammotheidae): arthropod ground pattern of gene arrangement. BMC Genomics 8: 343. 17908292 10.1186/1471-2164-8-343 PMC2194727

[19] Gissi C, Iannelli F, Pesole G (2008) Evolution of the mitochondrial genome of Metazoa as exemplified by comparison of congeneric species. Heredity 101: 301–320. 18612321 10.1038/hdy.2008.62

[20] Shao R, Barker SC, Mitani H, Takahashi M, Fukunaga M (2006) Molecular mechanisms for the variation of mitochondrial gene content and gene arrangement among chigger mites of the genus *Leptotrombidium* (Acari: Acariformes). J Mol Evol 63: 251–261. 16830100 10.1007/s00239-005-0196-y

[21] Jones M, Gantenbein B, Fet V, Blaxter M (2007) The effect of model choice on phylogenetic inference using mitochondrial sequence data: lessons from the scorpions. Mol Phylogenet Evol 43: 583–595. 17275351 10.1016/j.ympev.2006.11.017

[22] Choi EH, Park SJ, Jang KH, Hwang W (2007) Complete mitochondrial genome of a Chinese scorpion *Mesobuthus martensii* (Chelicerata, Scorpiones, Buthidae). DNA Seq 18: 461–473. 17676475 10.1080/10425170701289883

[23] Shao R, Barker SC, Mitani H, Aoki Y, Fukunaga M (2005) Evolution of duplicate control regions in the mitochondrial genomes of metazoa: a case study with Australasian *Ixodes* ticks. Mol Biol Evol 22: 620–629. 15537802 10.1093/molbev/msi047

[24] Black WC, Roehrdanz RL (1998) Mitochondrial gene order is not conserved in arthropods: prostriate and metastriate tick mitochondrial genomes. Mol Biol Evol 15: 1772–1785. 9866211 10.1093/oxfordjournals.molbev.a025903

[25] Gissi C, Pesole G (2003) Transcript mapping and genome annotation of ascidian mtDNA using EST data. Genome Res 13: 2203–2212. 12915488 10.1101/gr.1227803 PMC403730

[26] Berthier F, Renaud M, Alziari S, Durand R (1986) RNA mapping on *Drosophila* mitochondrial DNA: precursors and template strands. Nucleic Acids Res 14: 4519–4533. 3086843 10.1093/nar/14.11.4519 PMC311462

[27] Stewart JB, Beckenbach AT (2009) Characterization of mature mitochondrial transcripts in *Drosophila*, and the implications for the tRNA punctuation model in arthropods. Gene 445: 49–57. 19540318 10.1016/j.gene.2009.06.006

[28] Ojala D, Montoya J, Attardi G (1981) tRNA punctuation model of RNA processing in human mitochondria. Nature 290: 470–474. 7219536 10.1038/290470a0

[29] Schattner P, Brooks AN, Lowe TM (2005) The tRNAscan-SE, snoscan and snoGPS web servers for the detection of tRNAs and snoRNAs. Nucleic Acids Res 33: W686–689. 15980563 10.1093/nar/gki366 PMC1160127

[30] Laslett D, Canback B (2008) ARWEN: a program to detect tRNA genes in metazoan mitochondrial nucleotide sequences. Bioinformatics 24: 172–175. 18033792 10.1093/bioinformatics/btm573

[31] Smith C, Heyne S, Richter AS, Will S, Backofen R (2010) Freiburg RNA Tools: a web server integrating INTARNA, EXPARNA and LOCARNA. Nucleic Acids Res 38: W373–377. 20444875 10.1093/nar/gkq316 PMC2896085

[32] Zuker M (2003) Mfold web server for nucleic acid folding and hybridization prediction. Nucleic Acids Res 31: 3406–3415. 12824337 10.1093/nar/gkg595 PMC169194

[33] Betley JN, Frith MC, Graber JH, Choo S, Deshler JO (2002) A ubiquitous and conserved signal for RNA localization in chordates. Curr Biol 12: 1756–1761. 12401170 10.1016/s0960-9822(02)01220-4

[34] Grillo G, Licciulli F, Liuni S, Sbisa E, Pesole G (2003) PatSearch: A program for the detection of patterns and structural motifs in nucleotide sequences. Nucleic Acids Res 31: 3608–3612. 12824377 10.1093/nar/gkg548 PMC168955

[35] Pesole G, Liuni S, D’Souza M (2000) PatSearch: a pattern matcher software that finds functional elements in nucleotide and protein sequences and assesses their statistical significance. Bioinformatics 16: 439–450. 10871266 10.1093/bioinformatics/16.5.439

[36] Schneider T, Stephens R (1990) Sequence logos: A new way to display consensus sequences. Nucleic Acids Res 18: 6097–6100. 2172928 10.1093/nar/18.20.6097 PMC332411

[37] Crooks G, Hon G, Chandonia J, Brenner S (2004) WebLogo: a sequence logo generator. Genome Res 14: 1188–1190. 15173120 10.1101/gr.849004 PMC419797

[38] D’Onorio de Meo P, D’Antonio M, Griggio F, Lupi R, Borsani M, et al (2012) MitoZoa 2.0: a database resource and search tools for comparative and evolutionary analyses of mitochondrial genomes in Metazoa. Nucleic Acids Res 40: D1168–1172. 22123747 10.1093/nar/gkr1144 PMC3245153

[39] Lupi R, D’Onorio De Meo P, Picardi E, D’Antonio M, Paoletti D, et al (2010) MitoZoa: a curated mitochondrial genome database of metazoans for comparative genomics studies. Mitochondrion 10: 192–199. 20080208 10.1016/j.mito.2010.01.004

[40] Benkel BF, Duschesnay P, Boer PH, Genest Y, Hickey DA (1988) Mitochondrial large ribosomal RNA: an abundant polyadenylated sequence in *Drosophila* . Nucleic Acids Res 16: 9880. 3141905 10.1093/nar/16.20.9880 PMC338808

[41] Altschul SF, Gish W, Miller W, Myers EW, Lipman DJ (1990) Basic local alignment search tool. J Mol Biol 215: 403–410. 2231712 10.1016/S0022-2836(05)80360-2

[42] Drummond AJ, Ashton B, Buxton S, Cheung M, Cooper A, et al. (2010) Geneious v5.5.7 created by Biomatters. Available: http://www.geneious.com. Accessed 2010.

[43] Edgar RC (2004) MUSCLE: a multiple sequence alignment method with reduced time and space complexity. BMC Bioinformatics 5: 113. 15318951 10.1186/1471-2105-5-113 PMC517706

[44] Castresana J (2000) Selection of conserved blocks from multiple alignments for their use in phylogenetic analysis. Mol Biol Evol 17: 540–552. 10742046 10.1093/oxfordjournals.molbev.a026334

[45] Galtier N, Gouy M, Gautier C (1996) SEAVIEW and PHYLO_WIN: two graphic tools for sequence alignment and molecular phylogeny. Comput Appl Biosci 12: 543–548. 9021275 10.1093/bioinformatics/12.6.543

[46] Abascal F, Zardoya R, Posada D (2005) ProtTest: selection of best-fit models of protein evolution. Bioinformatics 21: 2104–2105. 15647292 10.1093/bioinformatics/bti263

[47] Posada D, Crandall KA (1998) MODELTEST: testing the model of DNA substitution. Bioinformatics 14: 817–818. 9918953 10.1093/bioinformatics/14.9.817

[48] Abascal F, Posada D, Zardoya R (2007) MtArt: a new model of amino acid replacement for Arthropoda. Mol Biol Evol 24: 1–5. 17043087 10.1093/molbev/msl136

[49] Lanave C, Preparata G, Saccone C, Serio G (1984) A new method for calculating evolutionary substitution rates. J Mol Evol 20: 86–93. 6429346 10.1007/BF02101990

[50] Huelsenbeck JP, Ronquist F (2001) MRBAYES: Bayesian inference of phylogenetic trees. Bioinformatics 17: 754–755. 11524383 10.1093/bioinformatics/17.8.754

[51] Adachi J, Hasegawa M (1996) Model of amino acid substitution in proteins encoded by mitochondrial DNA. J Mol Evol 42: 459–468. 8642615 10.1007/BF02498640

[52] Lavrov DV, Boore JL, Brown WM (2000) The complete mitochondrial DNA sequence of the horseshoe crab *Limulus polyphemus* . Mol Biol Evol 17: 813–824. 10779542 10.1093/oxfordjournals.molbev.a026360

[53] Shao R, Aoki Y, Mitani H, Tabuchi N, Barker SC, et al (2004) The mitochondrial genomes of soft ticks have an arrangement of genes that has remained unchanged for over 400 million years. Insect Mol Biol 13: 219–224. 15157222 10.1111/j.0962-1075.2004.00447.x

[54] Xu G, Fang QQ, Keirans JE, Durden LA (2003) Molecular phylogenetic analyses indicate that the Ixodes ricinus complex is a paraphyletic group. J Parasitol 89: 452–457. 12880241 10.1645/0022-3395(2003)089[0452:MPAITT]2.0.CO;2

[55] Klompen JSH, Black IV WC, Keirans JE, Norris DE (2000) Systematics and biogeography of hard ticks: a total evidence approach. Cladistics 16: 79–102. 10.1111/j.1096-0031.2000.tb00349.x 34902921

[56] Christianson TW, Clayton DA (1986) In vitro transcription of human mitochondrial DNA: accurate termination requires a region of DNA sequence that can function bidirectionally. Proc Natl Acad Sci USA 83: 6277–6281. 3018722 10.1073/pnas.83.17.6277 PMC386486

[57] Christianson TW, Clayton DA (1988) A tridecamer DNA sequence supports human mitochondrial RNA 3′-end formation in vitro. Mol Cell Biol 8: 4502–4509. 3185559 10.1128/mcb.8.10.4502-4509.1988 PMC365525

[58] Fernandez-Silva P, Martinez-Azorin F, Micol V, Attardi G (1997) The human mitochondrial transcription termination factor (mTERF) is a multizipper protein but binds to DNA as a monomer, with evidence pointing to intramolecular leucine zipper interactions. Embo J 16: 1066–1079. 9118945 10.1093/emboj/16.5.1066 PMC1169706

[59] Kruse B, Narasimhan N, Attardi G (1989) Termination of transcription in human mitochondria: identification and purification of a DNA binding protein factor that promotes termination. Cell 58: 391–397. 2752429 10.1016/0092-8674(89)90853-2

[60] Valverde JR, Marco R, Garesse R (1994) A conserved heptamer motif for ribosomal RNA transcription termination in animal mitochondria. Proc Natl Acad Sci U S A 91: 5368–5371. 7515499 10.1073/pnas.91.12.5368 PMC43996

[61] Fernandez-Silva P, Loguercio Polosa P, Roberti M, Di Ponzio B, Gadaleta MN, et al (2001) Sea urchin mtDBP is a two-faced transcription termination factor with a biased polarity depending on the RNA polymerase. Nucleic Acids Res 29: 4736–4743. 11713324 10.1093/nar/29.22.4736 PMC92518

[62] Loguercio Polosa P, Roberti M, Musicco C, Gadaleta MN, Quagliariello E, et al (1999) Cloning and characterisation of mtDBP, a DNA-binding protein which binds two distinct regions of sea urchin mitochondrial DNA. Nucleic Acids Res 27: 1890–1899. 10101198 10.1093/nar/27.8.1890 PMC148398

[63] Roberti M, Mustich A, Gadaleta MN, Cantatore P (1991) Identification of two homologous mitochondrial DNA sequences, which bind strongly and specifically to a mitochondrial protein of *Paracentrotus lividus* . Nucleic Acids Res 19: 6249–6254. 1956785 10.1093/nar/19.22.6249 PMC329135

[64] Roberti M, Loguercio Polosa P, Bruni F, Musicco C, Gadaleta MN, et al (2003) DmTTF, a novel mitochondrial transcription termination factor that recognises two sequences of *Drosophila melanogaster* mitochondrial DNA. Nucleic Acids Res 31: 1597–1604. 12626700 10.1093/nar/gkg272 PMC152874

[65] Van Etten RA, Bird JW, Clayton DA (1983) Identification of the 3′-ends of the two mouse mitochondrial ribosomal RNAs. The 3′-end of 16S ribosomal RNA contains nucleotides encoded by the gene for transfer RNALeuUUR. J Biol Chem 258: 10104–10110. 6309767

[66] Campbell NJH, Barker SC (1999) The novel mitochondrial gene arrangement of the cattle tick, *Boophilus microplus*: fivefold tandem repetition of a coding region. Mol Biol Evol 16: 732–740. 10368952 10.1093/oxfordjournals.molbev.a026158

[67] Shao R, Mitani H, Barker SC, Takahashi M, Fukunaga M (2005) Novel mitochondrial gene content and gene arrangement indicate illegitimate inter-mtDNA recombination in the chigger mite, *Leptotrombidium pallidum* . J Mol Evol 60: 764–773. 15931495 10.1007/s00239-004-0226-1

[68] Pesole G, Gissi C, De Chirico A, Saccone C (1999) Nucleotide substitution rate of mammalian mitochondrial genomes. J Mol Evol 48: 427–434. 10079281 10.1007/pl00006487

[69] Friedrich M, Tautz D (1997) Evolution and phylogeny of the Diptera: a molecular phylogenetic analysis using 28S rDNA sequences. Systematic Biology 46: 674–698. 11975338 10.1093/sysbio/46.4.674

[70] Krzeminski W, Krzeminska E (2003) Triassic Diptera: description, revisions, and phylogenetic relations. Acta Zoologica Cracoviensia 46 Supp: 153–184

[71] Roberti M, Bruni F, Loguercio Polosa P, Gadaleta MN, Cantatore P (2006) The *Drosophila* termination factor DmTTF regulates in vivo mitochondrial transcription. Nucleic Acids Res 34: 2109–2116. 16648357 10.1093/nar/gkl181 PMC1450328

[72] Montooth KL, Abt DN, Hofmann JW, Rand DM (2009) Comparative genomics of *Drosophila* mtDNA: Novel features of conservation and change across functional domains and lineages. J Mol Evol 69: 94–114. 19533212 10.1007/s00239-009-9255-0 PMC2895726

[73] Roberti M, Loguercio Polosa P, Bruni F, Manzari C, Deceglie S, et al (2009) The MTERF family proteins: mitochondrial transcription regulators and beyond. Biochim Biophys Acta 1787: 303–311. 19366610 10.1016/j.bbabio.2009.01.013

[74] Linder T, Park CB, Asin-Cayuela J, Pellegrini M, Larsson NG, et al (2005) A family of putative transcription termination factors shared amongst metazoans and plants. Curr Genet 48: 265–269. 16193327 10.1007/s00294-005-0022-5

[75] Munderloh UG, Liu Y, Wang M, Chen C, Kurtti TJ (1994) Establishment, maintenance and description of cell lines from the tick *Ixodes scapularis* . J Parasitol 80: 533–543. 8064520

[76] Najm N-A, Silaghi C, Bell-Sakyi L, Pfister K, Passos L (2012) Detection of bacteria related to "Candidatus" *Midichloria mitochondrii* in tick cell lines. Parasitology Research 110: 437–442. 21748354 10.1007/s00436-011-2509-y

[77] Hixson JE, Wong TW, Clayton DA (1986) Both the conserved stem-loop and divergent 5′-flanking sequences are required for initiation at the human mitochondrial origin of light-strand DNA replication. J Biol Chem 261: 2384–2390. 3944140

[78] Macey JR, Larson A, Ananjeva NB, Fang Z, Papenfuss TJ (1997) Two novel gene orders and the role of light-strand replication in rearrangement of the vertebrate mitochondrial genome. Mol Biol Evol 14: 91–104. 9000757 10.1093/oxfordjournals.molbev.a025706

[79] Murrell A, Barker SC (2003) Synonymy of Boophilus Curtice, 1891 with Rhipicephalus Koch, 1844 (Acari: Ixodidae). Syst Parasitol 56: 169–172. 14707501 10.1023/b:sypa.0000003802.36517.a0

[80] Meusemann K, von Reumont BM, Simon S, Roeding F, Strauss S, et al (2010) A phylogenomic approach to resolve the arthropod tree of life. Mol Biol Evol 27: 2451–2464. 20534705 10.1093/molbev/msq130

