# Supplementary material for: Tick-Box for 3′-End Formation of Mitochondrial Transcripts in Ixodida, Basal Chelicerates and Drosophila
Source: PLoS One. 2012 Oct 15;7(10):e47538. doi: 10.1371/journal.pone.0047538 (PMC3471875; doi:10.1371/journal.pone.0047538)
Supplement: Figure S2 — Conserved motifs and secondary structures of the tick control region, mapped on the I. ricinus sequence. (PDF) [file pone.0047538.s002.pdf]

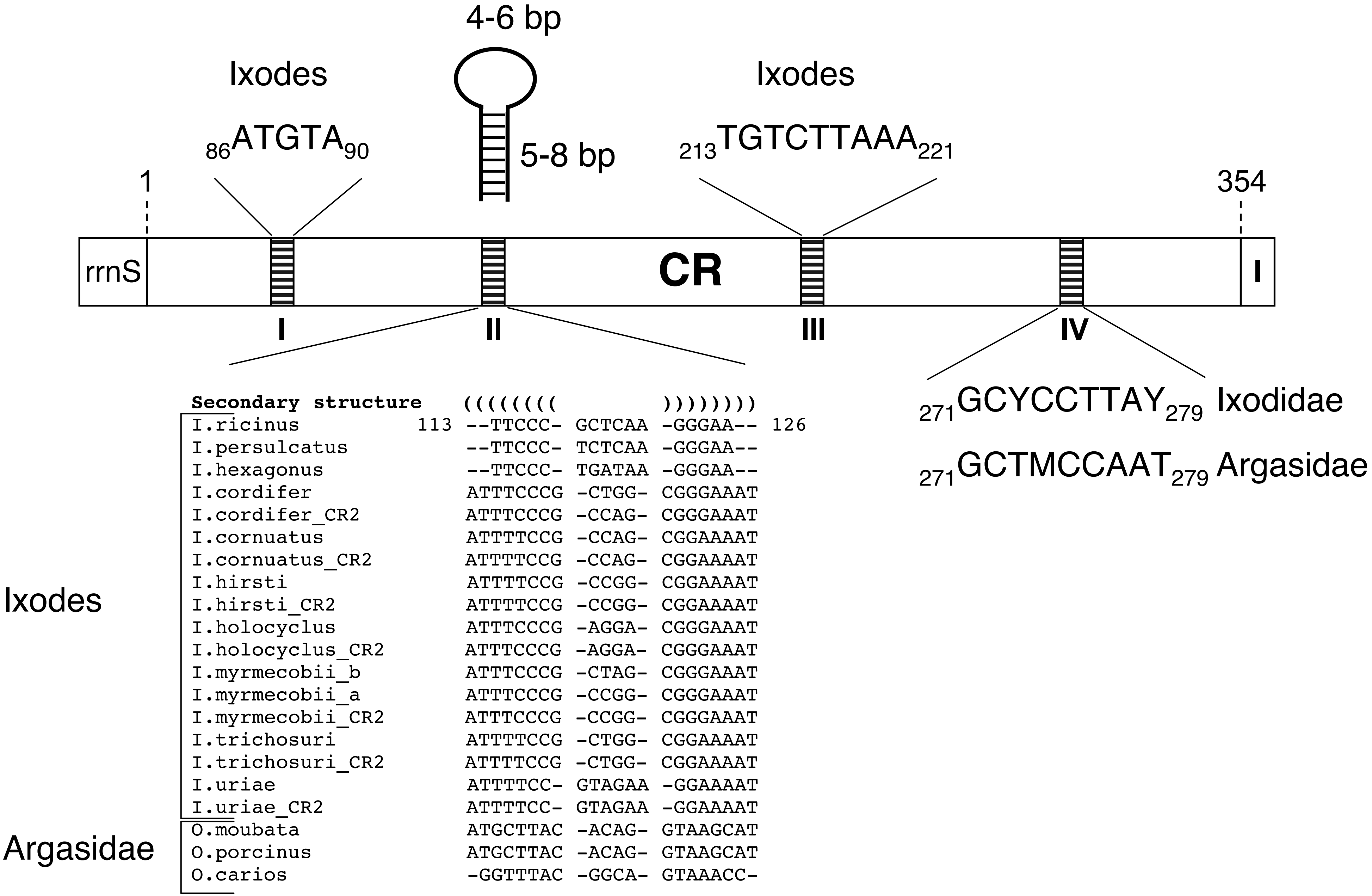
4-6 bp
5-8 bp
Ixodes
86ATGTA90
Ixodes
213TGTCTTAAA221
1
354
rrnS
CR
I
I
II
III
IV
271GCYCCTTAY279 Ixodidae
271GCTMCCAAT279 Argasidae
Secondary structure (((((((( ))))))))
I.ricinus 113 --TTCCC- GCTCAA -GGGAA-- 126
I.persulcatus --TTCCC- TCTCAA -GGGAA--
I.hexagonus --TTCCC- TGATAA -GGGAA--
I.cordifer ATTTCCCG -CTGG- CGGGAAAT
I.cordifer_CR2 ATTTCCCG -CCAG- CGGGAAAT
I.cornuatus ATTTTCCG -CCAG- CGGAAAAT
I.cornuatus_CR2 ATTTTCCG -CCAG- CGGAAAAT
I.hirsti ATTTTCCG -CCGG- CGGAAAAT
I.hirsti_CR2 ATTTTCCG -CCGG- CGGAAAAT
I.holocyclus ATTTCCCG -AGGA- CGGGAAAT
I.holocyclus_CR2 ATTTCCCG -AGGA- CGGGAAAT
I.myrmecobii_b ATTTCCCG -CTAG- CGGGAAAT
I.myrmecobii_a ATTTCCCG -CCGG- CGGGAAAT
I.myrmecobii_CR2 ATTTCCCG -CCGG- CGGGAAAT
I.trichosuri ATTTTCCG -CTGG- CGGAAAAT
I.trichosuri_CR2 ATTTTCCG -CTGG- CGGAAAAT
I.uriae ATTTTCC- GTAGAA -GGAAAAT
I.uriae_CR2 ATTTTCC- GTAGAA -GGAAAAT
O.moubata ATGCTTAC -ACAG- GTAAGCAT
O.porcinus ATGCTTAC -ACAG- GTAAGCAT
O.carios -GGTTTAC -GGCA- GTAAACC-
Ixodes
Argasidae
