# Supplementary material for: Tick-Box for 3′-End Formation of Mitochondrial Transcripts in Ixodida, Basal Chelicerates and Drosophila
Source: PLoS One. 2012 Oct 15;7(10):e47538. doi: 10.1371/journal.pone.0047538 (PMC3471875; doi:10.1371/journal.pone.0047538)
Supplement: Text S1 — Amplification strategy and general features of tRNAs, control region, and small non-coding regions of the I. ricinus mtDNA. (DOC) [file pone.0047538.s004.doc]

**SUPPORTING INFORMATION of**

**Tick-Box for 3’-end formation of mitochondrial transcripts in Ixodida, basal chelicerates and *Drosophila***

Matteo Montagna ^1^, Davide Sassera ^1^, Francesca Griggio ^2^, Sara Epis ^1^, Claudio Bandi ^1^, Carmela Gissi ^2^*

1 Università degli Studi di Milano, Dipartimento di Scienze Veterinarie e Sanità Pubblica, Milano, Italy

2 Università degli Studi di Milano, Dipartimento di Bioscienze, Milano, Italy

* Corresponding author:

Carmela Gissi

carmela.gissi@unimi.it

Università degli Studi di Milano, Dipartimento di Bioscienze

Via Celoria 26, 20133 Milano, Italy

**This file includes Tables S2-S3 and Legend of Figure S1-S2**

**MATERIALS AND METHODS**

***Tick collection and mtDNA amplification***

An adult female of *Ixodes ricinus* was collected from Monte Cornizzolo (Como, Italy). The sample was washed in distilled water and heated at 95°C for 5 minutes in lysis buffer (Qiagen) to inactivate DNAses. DNA extraction was performed with the DNeasy Blood & Tissue Kit (Qiagen).

The whole mtDNA of *I. ricinus* was amplified in 11n overlapped fragments, ranging from 350 to 5500 bp, using 17 mitochondrial-specific primers (Table S2) designed on the available sequences of other *Ixodes* species. These primers were used in several combinations in both standard and long PCR reactions using the GoTaq (Promega) and the Crimson LongAmp *Taq* (New England Biolabs), respectively. Successful single-band amplifications were sequenced directly or by primer walking, according to the Sanger method and using an Applied Biosystems Sequencer. Single reads were manually checked and assembled with gap4 [1]. Ambiguous mt regions were confirmed by additional PCRs.

**RESULTS AND DISCUSSION**

***General Features of the I. ricinus mitochondrial genome***

The mtDNA of *I. ricinus* is 14,566 bp long, thus comparable in length to the mtDNA of other Ixodida (Table S3). The genome is AT-rich, with a 78.7% AT-content similar to that of other Ixodida (average AT% = 75.2± 2.7 %) (Table S3). The high AT-content is reflected in codon usage by the preference towards the usage of A and T at the third position of synonymous codons, and by the abundance of AUU (Ile) and UUU (Phe) codons (409 and 380 occurrences, respectively).

***Peculiarities of overlapped protein-coding genes***

Both the *atp8*/*atp6* and *nad4*/*nad4L* gene pairs of *I. ricinus* exhibit a 7 bp overlap, thus supporting the synthesis of a mature bicistronic mRNA for each of these gene pairs [2,3]. The reason for conservation of a heptamer as overlap sequence is obscure, although we can hypothesize the existence of functional constraints related to the translation of a bicistronic mRNA. Surprisingly, the size and sequence of this overlap is conserved for both these gene pairs in all 11 analysed ticks (Table 1), with nucleotide (nt) differences observed only at the fourth and at the last position of the overlapped sequence (consensus heptamer: ATGATAr in *atp8*/*atp6;* ATGyTAr in *nad4*/*nad4L;* ATGhTAr in the two gene pairs together). The occurrence of nt substitutions at only these two positions can be explained considering that the heptamer is subjected to the functional constraints of two different ORFs. Indeed, the first nt triplet needs to be conserved since it is the start codon of the second ORF of the bicistronic mRNA (*atp6* and *nad4L*); the last nt triplet needs to be conserved as TAr because it is the stop codon of the first ORF of the bicistronic mRNA (*atp8* and *nad4*); finally, the fourth position of the heptamer behaves as a first or a third codon position, depending on the ORF considered, thus it can more easily tolerate nt substitutions.

***Transfer RNAs***

The tRNA genes of *I. ricinus* range in size from 56 to 70 bp, and show the typical cloverleaf secondary structure of animal mt tRNAs [4] (Figure S1). As expected, only *trnS(AGN)* lacks of DHU arm. The *trnC* gene, deficient of the DHU arm in metastriata [5], has the canonical four-arm cloverleaf structure found in all other Prostriata and Argasidae ticks.

As shown in Figure S1, the anticodon (AC) sequence is preceded by a T and followed by an A in all tRNAs except for *trnE*, *trnH* and *trnL(UUR)*. In these three tRNAs, the anticodon is followed by a G. The stems of DHU and T arms are quite variable in length, being their size 3-4 and 2-5 bp, respectively. This length variability is very similar to that observed in the variable loops, ranging from 2 to 5 bp. The AC and the amino acid acceptor (AA) stems have a constant size of 7 and 6 bp, respectively, in accordance with the animal tRNA model proposed by Kumazawa and Nishida [4]. In total, only eight unpaired base pairs have been found in the stem regions of all *I. ricinus* tRNAs. Indeed, single mismatched base pairs are present in: the AC stem of *trnK*, *trnM* and *trnT*; the DHU stem of *trnC* and *trnI*; the T stem of *trnD* and *trnL(UUR)*; and the first position of the AA stem of *trnM*. Since the mispairing in the AA stem of *trnM* is present in all *Ixodes* species, a short *trnM* gene with an AA stem of only 6 bp has been previously annotated in the *Ixodes* species. However, it should be noted that the first position of the AA stem is well paired in the *trnM* of all Metastriata and of one Argasidae species, and that one mispairing is tolerated in the stem of functional tRNAs [4,6]. Consequently, we have annotated the *trnM* of *I. ricinus* with a canonical 7 bp AA arm.

The comparison of *I. ricinus* tRNAs with the homologous genes of other 4 *Ixodes* species (Table 1 in the main text) shows that the loops of DHU and T arms are the most variable tRNA regions, both in sequence and length. In general, indels and nt substitutions are very frequently in these loops, while compensatory substitutions are prevalent in the stem regions. Moreover, most nt substitutions in tRNAs clearly distinguish Australasian from non-Australasian *Ixodes*.

***The control region***

The longest non-coding region of arthropods is often indicated as control region (CR), as it usually contains the regulatory elements for mtDNA replication and transcription. The CR of *I. ricinus* is 354 bp long and is located between *rrnS* and *trnI*, the ancestral position of arthropods [7,8,9]. As shown in Table S3, its size is comparable to the CR of other ticks and to the duplicated CR2 of Australasian *Ixodes* and Metastriata. Although the CR is often reported as the most AT-rich region of the arthropod mtDNA, the CR of *I. ricinus* shows almost the same AT% of the whole mt genome (Table S3). This observation holds for almost all Ixodida, except two metastriates whose CR and CR2 have an AT% even lower than that of the entire mtDNA (*Rhipicephalus* and *Haemaphisalis* in Table S3). Therefore, the CRs of ticks do not follow the typical trend of other arthropods towards an increase of AT%, and in Ixodida the term “AT-rich region” should not be used as synonymous of CR.

The CR of *I. ricinus* was aligned and compared to the CR and CR2 fully sequenced in 15 tick species (Table S1), allowing identifying four sequence motifs, including one hairpin structure conserved in some taxa (Figure S2). Motifs I and III (Figure S2) are short sequences perfectly conserved in all analysed *Ixodes* species and were identified by Shao et al. [10] as specific of Australasian *Ixodes*. Motif IV is a 9 bp C-rich sequence present in the CR and CR2 of all analysed tick species, although with a different consensus depending on the taxonomic group (Figure S2). Finally, motif II is a hairpin secondary structure with a 5-8 bp stem and a loop of 4-6 bp. In *I. ricinus*, this hairpin is a stable structure with a ΔG = -2.93 kcal/mol (MFold). This secondary structure is conserved only in *Ixodes* and Argasidae (Mfold analyses), and encompasses two close elements identified as specific of Australasian *Ixodes* by Shao et al. [10].

The conservation of these motifs indicates that they may have specific roles in the tick mtDNA replication and/or transcription. It should be noted that several Authors have hypothesized the association of hairpin structures to the mtDNA replication origin(s) [11,12].

***Small non-coding regions***

The *I. ricinus* mtDNA has only 11 small NCRs, for a total of 72 bp corresponding to 0.49% of the entire mtDNA (Table S3). These values are similar to those observed in other Ixodida, except for *R. sanguineus* and Argasidae, where the number and length of single NCRs increase and drastically decrease, respectively (Table S3).

A detailed analysis of the non-coding regions (excluding the CR and the Tick-Box) shows that most tick NCRs are 1-6 bp long and have a different length from one species to the other, thus they can be considered as simple gene spacers with no specific functions. As an exception, the NCR between *trnL(UUR)* and *trnL(CUN)* ranges from 9 to 14 bp in *Ixodes* species, is 2-4 bp long in Argasidae, and is absent in Metastriata due to the gene order rearrangement (Figure 1 of the main text). Thus, this NCR is an AT-rich sequence (AT% >83%) showing a tendency to size variation related to the taxonomic group.

In the three complete mtDNAs of Metastriata, the NCR between *trnQ* and *trnF* is 9-25 bp long but its size ranges from 3 to 36 bp when partial sequences are also considered (Table S1). As reported in the main paper, this NCR is located in a gene adjacency restricted only to Metastriata. Moreover, in some species it includes a third copy of the Tick-Box motif in the reverse-complement orientation compared to the Tick-box motifs downstream of *nad1* and *rrnL*.

On the overall, these analyses show that the mtDNA of *I. ricinus* conforms to that of other *Ixodes* species in size and distribution of small NCRs. Moreover, there are no small conserved NCRs other than those containing the Tick-Box.

**REFERENCES**

1. Staden R, Beal KF, Bonfield JK (2000) The Staden package, 1998. Methods Mol Biol 132: 115-130.

2. Berthier F, Renaud M, Alziari S, Durand R (1986) RNA mapping on *Drosophila* mitochondrial DNA: precursors and template strands. Nucleic Acids Res 14: 4519-4533.

3. Stewart JB, Beckenbach AT (2009) Characterization of mature mitochondrial transcripts in *Drosophila*, and the implications for the tRNA punctuation model in arthropods. Gene 445: 49-57.

4. Kumazawa Y, Nishida M (1993) Sequence evolution of mitochondrial tRNA genes and deep-branch animal phylogenetics. J Mol Evol 37: 380-398.

5. Shao R, Aoki Y, Mitani H, Tabuchi N, Barker SC, et al. (2004) The mitochondrial genomes of soft ticks have an arrangement of genes that has remained unchanged for over 400 million years. Insect Mol Biol 13: 219-224.

6. Watanabe K (2010) Unique features of animal mitochondrial translation systems. The non-universal genetic code, unusual features of the translational apparatus and their relevance to human mitochondrial diseases. Proc Jpn Acad Ser B Phys Biol Sci 86: 11-39.

7. Lavrov DV, Boore JL, Brown WM (2000) The complete mitochondrial DNA sequence of the horseshoe crab *Limulus polyphemus*. Mol Biol Evol 17: 813-824.

8. Boore JL, Lavrov DV, Brown WM (1998) Gene translocation links insects and crustaceans. Nature 392: 667-668.

9. Boore JL, Collins TM, Stanton D, Daehler LL, Brown WM (1995) Deducing the pattern of arthropod phylogeny from mitochondrial DNA rearrangements. Nature 376: 163-165.

10. Shao R, Barker SC, Mitani H, Aoki Y, Fukunaga M (2005) Evolution of duplicate control regions in the mitochondrial genomes of metazoa: a case study with Australasian *Ixodes* ticks. Mol Biol Evol 22: 620-629.

11. Clary DO, Wolstenholme DR (1987) *Drosophila* mitochondrial DNA: Conserved sequences in the A + T -rich region and supporting evidence for a secondary structure model of the small ribosomal RNA J Mol Evol 25: 116-125.

12. Zhang DX, Szymura JM, Hewitt GM (1995) Evolution and structural conservation of the control region of insect mitochondrial DNA. J Mol Evol 40: 382-391.

**Table S2.** Primers used to analyse the mitochondrial genome of *Ixodes ricinus.*

| **Primer ^a^** | **Sequence (5’ -> 3’)** | **Usage** |
| --- | --- | --- |
| IRM_11857 | AAAGCAACTCTTACTAAAACAC | PCR/Sequencing |
| IRM_13741 | TTAGATACCCTATTATTTTAAGC | PCR/Sequencing |
| IRM_10310 | AGTTGATAATAATACACTCAC | PCR/Sequencing |
| IRM_11862 | CTTTGTGTTTTAGTAAGAGTT | PCR/Sequencing |
| IRM_11857 | AAAGCAACTCTTACTAAAACAC | PCR/Sequencing |
| IRM_1250 | TTCCAATGTCTTTATGGTTAGTAG | PCR/Sequencing |
| IRM_3303 | TTTTCCTTTGCTTCACGC | PCR/Sequencing |
| IRM_4988 | GWHCCAAAAATTCTGTCTCT | PCR/Sequencing |
| IRM_4833 | TAATCTCTTCAGGDATTTCA | PCR/Sequencing |
| IRM_6776 | TTATTTTTATGCGCGGGGTTA | PCR/Sequencing |
| IRM_8690 | AAATHCCCGCTTGTAAACG | PCR/Sequencing |
| IRM_11862 | CTTTGTGTTTTAGTAAGAGTT | PCR/Sequencing |
| IRM_10312 | TCGAGTGTATTATTATCAACTG | PCR/Sequencing |
| IRM_13364 | ATGTTACGACTTATCTCACGG | PCR/Sequencing |
| IRM_149 | TTCTAAGGATATTCATAGGGC | PCR/Sequencing |
| IRM_1380 | GGTTGTCCTAATTCAGTTCG | PCR/Sequencing |
| IRM_1252 | CTACTAACCATAAAGACATTGG | PCR/Sequencing |
| nad1_173pr | TTTCAACCTTTAAGAGATGCTGT | 3’RACE |
| nad1_620pr | CGTAGTCCATTTGATTTAACTGA | 3’RACE |
| rrnL_850pr | AAATTAGGGACAAGAAGACC | 3’RACE |
| rrnL_1050pr | AATACTCTAGGGATAACAGCGT | 3’RACE |

a: in the sequencing primers, numbers refer to the position on the mtDNA of *Ixodes persulcatus*; in 3’ RACE primers, numbers refer to the position on the corresponding gene of *I. ricinus*

**Table S3.** Size and base composition of mtDNA, control region (CR), and small non-coding regions (NCR) of Ixodida.

|  | **mtDNA** | | **CR** | | **CR2** | | **Small NCR** | | |
| --- | --- | --- | --- | --- | --- | --- | --- | --- | --- |
|  | **bp** | **AT%** | **bp** | **AT%** | **bp** | **AT%** | **N°** | **bp** | **%** |
| *Ixodes ricinus* | 14566 | 78.6 | 354 | 78.5 | none |  | 11 | 72 | 0.49 |
| *Ixodes hexagonus* | 14539 | 72.7 | 358 | 71.9 | none |  | 15 | 82 | 0.56 |
| *Ixodes persulcatus* | 14539 | 77.3 | 352 | 77.6 | none |  | 12 | 79 | 0.54 |
| *Ixodes holocyclus* | 15007 | 77.4 | 352 | 78.4 | 450 | 80.0 | 14 | 93 | 0.62 |
| *Ixodes uriae* | 15053 | 74.8 | 388 | 77.1 | 476 | 71.0 | 14 | 93 | 0.62 |
| *Amblyomma triguttatum* | 14740 | 78.4 | 307 | 71.6 | 307 | 71.7 | 14 | 89 | 0.60 |
| *Haemaphisalis flava* | 14686 | 76.9 | 310 | 66.8 | 310 | 66.5 | 14 | 76 | 0.52 |
| *Rhipicephalus sanguineus* | 14710 | 78.0 | 304 | 66.6 | 303 | 67.3 | 18 | 130 | 0.88 |
| *Carios capensis* | 14418 | 73.5 | 342 | 71.4 | none |  | 9 | 40 | 0.28 |
| *Ornithodoros moubata* | 14398 | 72.3 | 342 | 71.6 | none |  | 6 | 37 | 0.26 |
| *Ornithodoros porcinus* | 14378 | 71.0 | 338 | 69.5 | none |  | 6 | 37 | 0.26 |

CR: ancestral control region; CR2: duplicated control region

**FIGURE LEGEND**

**Figure S1.** Putative secondary structure of the 22 tRNAs of *I. ricinus*. Lower case indicates positions with nucleotide substitutions in the homologous tRNAs of the other 4 analysed *Ixodes* species (Table 1 of the main paper). Substitutions present in the DHU, T and variable loops are not reported. Canonical and G:T base pairs are differently indicated.

**Figure S2.** Conserved motifs and secondary structures of the tick control region, mapped on the *I. ricinus* sequence.

Dashed blocks indicate conserved motifs/secondary structures, with numbers referring to their position in the *I. ricinus* control region. The two tandem repeats of *Ixodes myrmecobii* are indicated as “_a” and “_b”. Gene abbreviations are reported as in Figure 1 of the main paper. Accession number of the analysed sequences is reported in Table S1.
