# Supplementary material for: Tick-Box for 3′-End Formation of Mitochondrial Transcripts in Ixodida, Basal Chelicerates and Drosophila
Source: PLoS One. 2012 Oct 15;7(10):e47538. doi: 10.1371/journal.pone.0047538 (PMC3471875; doi:10.1371/journal.pone.0047538)
Supplement: Figure S1 — Putative secondary structure of the 22 tRNAs of I. ricinus . (PDF) [file pone.0047538.s001.pdf]

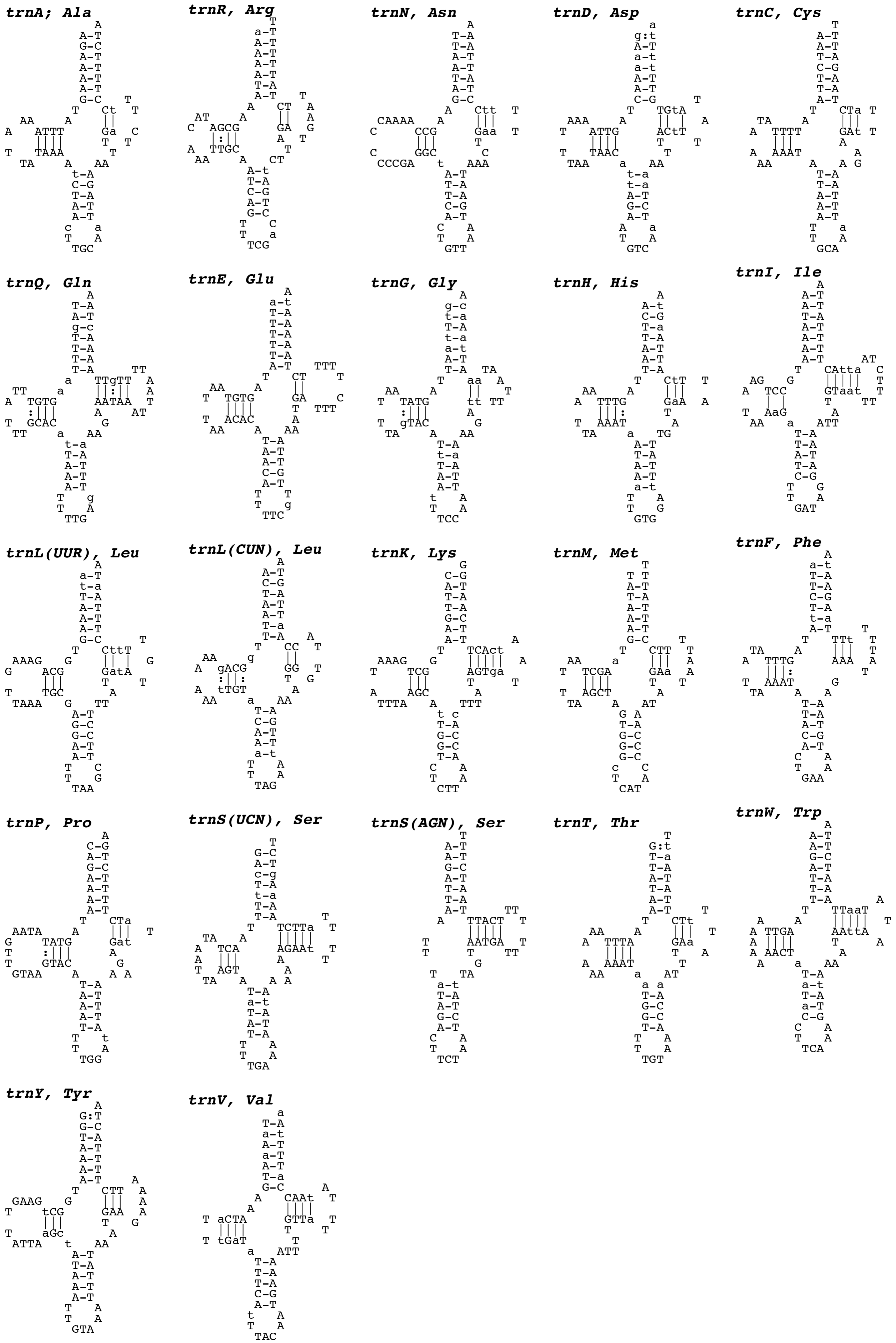

trnA; Ala
trnR, Arg
trnN, Asn
trnD, Asp
trnC, Cys
trnQ, Gln
trnE, Glu
trnG, Gly
trnH, His
trnI, Ile
trnL(UUR), Leu
trnL(CUN), Leu
trnK, Lys
trnM, Met
trnF, Phe
trnP, Pro
trnS(UCN), Ser
trnS(AGN), Ser
trnT, Thr
trnW, Trp
trnY, Tyr
trnV, Val
